# Metaflammation’s Role in Systemic Dysfunction in Obesity: A Comprehensive Review

**DOI:** 10.3390/ijms262110445

**Published:** 2025-10-27

**Authors:** Ioana-Maria Crasan, Matei Tanase, Corina Elena Delia, Gratiela Gradisteanu-Pircalabioru, Anisoara Cimpean, Elena Ionica

**Affiliations:** 1 Faculty of Biology, University of Bucharest, 91-95 Splaiul Independentei, 5th District, 050095 Bucharest, Romania; ioana.crasan@s.unibuc.ro (I.-M.C.); tanase.matei22@s.bio.unibuc.ro (M.T.); corina-elena.delia@unibuc.ro (C.E.D.); gratiela.gradisteanu@bio.unibuc.ro (G.G.-P.); anisoara.cimpean@bio.unibuc.ro (A.C.); 2 National Institute for Mother and Child Health “Alessandrescu-Rusescu” (NIMCH “Alessandrescu-Rusescu”), 120 Lacul Tei Boulevard, 2nd District, 020395 Bucharest, Romania

**Keywords:** obesity, metaflammation, endotoxemia, macrophages, gut microbiota, gut hormones

## Abstract

Obesity is redefined as a complex systemic disease, transcending mere caloric imbalance, driven by intricate dysregulation across metabolic, neuroendocrine, immunological, and epigenetic axes. Central to its pathology is adipose tissue, which is considered a dynamic endocrine and immune organ. Its dysfunctional expansion fuels chronic, low-grade systemic inflammation, termed “metaflammation”, characterised by pathways such as NF-kB and NLRP3 inflammasome activation, as well as pervasive immune cell infiltration. This inflammatory state could profoundly impair insulin signalling and contribute to major complications, including insulin resistance, type 2 diabetes, and cardiovascular disease. Further exacerbating this systemic dysfunction is gut microbiota dysbiosis, which promotes metabolic endotoxemia and neuroendocrine dysregulation, impacting hypothalamic function, central hormone resistance, and reproductive health. Epigenetic modifications also serve as crucial mediators, translating environmental exposures into altered gene expression that perpetuates susceptibility across generations. This review summarises the current understanding of obesity by integrating molecular, neuroendocrine, and immunometabolic underpinnings, reinterpreting it as a comprehensive expression of systemic dysfunction. Through this integrated perspective our hope is to highlight the necessity of a paradigm shift towards personalised, multi-targeted interventions that extend beyond conventional weight management. An integrative, translational approach modulating the immunometabolic network, microbiota, and epigenetics is essential to effectively address the global obesity epidemic and its far-reaching health implications.

## 1. Introduction: Redefining Obesity as a Systemic Dysfunction

### 1.1. Various Types and Key Contributing Axes

Obesity is no longer merely seen as a mismatch between the calories consumed and the energy expended. Recent biomedical research has increasingly characterised the illness as a result of a multidimensional systemic dysfunction, in which fatty tissues become dynamic organs within multiple interacting systems of neuroendocrine axes and the immune system, playing central roles in maintaining organismal homeostasis [1]. This comprehensive understanding of obesity as a multifaceted disease, involving intricate interactions between genetic predispositions, epigenetic modifications, environmental factors, and systemic inflammation, underscores the necessity for personalised and multi-pronged therapeutic strategies.

Adipose tissues are recognised to be involved not only in storing energy but also in communicating through highly complex networks of adipokines, cytokines, and bioactive mediators with the nervous, immune, and metabolic systems [2,3,4]. Enlarging, however, pathologically adipose tissues give rise to the creation of states of low-grade, chronic inflammatory processes, further sustained by factors such as hypoxia, endoplasmic reticulum stress, and immune cell infiltration [1,5,6].

Apart from lifestyle and nutritional habits, genetic and epigenetic factors have been shown to influence an individual’s susceptibility to obesity and their variable response to therapeutic interventions [7]. New evidence suggests that intrauterine exposure to an obesogenic environment, characterised by maternal hypernutrition and gestational inflammation, could induce lasting epigenetic alterations in offspring, thereby predisposing them to obesity and related metabolic disorders in later life [8]. Such early-life epigenetic modifications, including DNA methylation and histone acetylation patterns, can program metabolic pathways and influence adipogenesis and energy expenditure throughout the lifespan [9]. Furthermore, the intricate interplay between genetics and environmental factors, coupled with the dynamic nature of epigenetic tags, contributes to the observed phenotypic variations in obesity [10].

Additionally, it has been observed that obesity manifests as the biological outcome of the persistent adversities posed by obesogenic environments, socioeconomic stress, chronic psychological stress, and an ineffective neuroendocrine reward system, which leads to the malfunction of over-the-counter energy balance regulation [1,11,12]. This dysbiosis of microbiota has been shown to worsen the situation in terms of metabolic abnormalities due to a disturbed *Firmicutes*/*Bacteroidetes* ratio, along with decreased formation of short-chain fatty acids and an increase in systemic endotoxemia, all triggering widespread dysregulation in immunometabolic state [11,13].

Increasing evidence suggests that the pathophysiology of obesity involves neuroinflammation, hypothalamic dysfunction, and significant endocrine dysfunction, including the regulation of leptin (LEP), ghrelin, insulin, and reproductive hormones [5,14,15,16,17]. On the other hand, has been reported that metabolic inflammation expands its scope beyond the liver, muscle, and pancreas to immune-privileged targets such as the brain and gonads, manifesting as a fully systemic disease [18].

**Figure 1:**
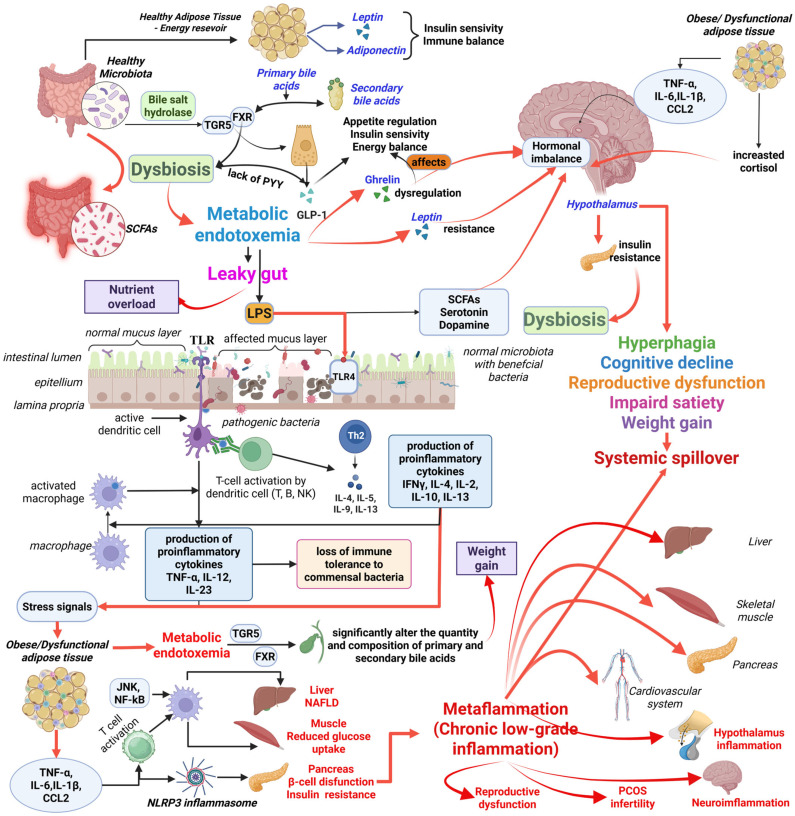
Metaflammation’s role in systemic dysfunction in obesity. This figure illustrates the contrasting states of a healthy gut–brain axis and a dysbiotic, pro-inflammatory state associated with obesity. In a healthy condition (top left), diverse gut microbiota ferment dietary fibers into short-chain fatty acids (SCFA), which support colonocyte health, preserve gut barrier integrity, and regulate immunity. These bacteria also convert primary bile acids into secondary bile acids, activating Takeda G-protein-coupled receptor 5 (TGR5)/farnesoid X receptor (FXR) that boost Glucagon-like peptide-1 (GLP-1) release and improve insulin sensitivity, thus strengthening the gut barrier and normal hormone signalling. Conversely (middle left), gut dysbiosis deteriorates intestinal barrier function, permitting bacterial lipopolysaccharides (LPS) to enter the bloodstream. This endotoxemia activates Toll-like receptor 4 (TLR4) on immune cells, triggering Nuclear Factor kappa-light-chain-enhancer of activated B cells (NF-kB) and releasing pro-inflammatory cytokines, leading to chronic, low-grade systemic inflammation (“metaflammation”). These inflammatory signals and disrupted gut hormones impair hypothalamic regulation of appetite and metabolism (top right), causing hyperphagia, insulin and leptin resistance, and weight gain. The diagram highlights how circulating hormones (leptin, insulin, ghrelin) and inflammatory signals inform the brain about metabolic status, affecting satiety, overeating, cognitive decline, reproductive issues, and obesity, often resulting in organ-specific pathologies in chronic endotoxemia/metaflammation. As a consequence of “systemic spillover”, organs such as the liver, skeletal muscle, pancreas, cardiovascular system, and reproductive organs exhibit pathologies (e.g., Non-alcoholic fatty liver disease (NAFLD), muscle insulin resistance, pancreatic β-cell dysfunction, atherogenesis, Polycystic ovary syndrome (PCOS) due to persistent inflammation.

Based on the literature review synthesising existing research on obesity, Table 1 provides a general overview of the complex and interconnected nature of obesity as a systemic disease, affecting numerous bodily functions at both molecular and physiological levels. Both outline the primary ways obesity causes dysfunction in the neuroendocrine, immune, metabolic, epigenetic, and microbiome systems, along with their associated effects. For instance, it is shown how leptin resistance and hypothalamic inflammation impair neuroendocrine functions, resulting in appetite disturbances and reduced fertility. The table also explains how chronic low-grade inflammation and M1 macrophage infiltration contribute to the development of insulin resistance and type 2 diabetes (T2DM). Additionally, it details how metabolic problems, such as lipotoxicity and mitochondrial dysfunction, could cause hepatic steatosis, while epigenetic changes and gut bacterial imbalances are linked to inherited risks and widespread inflammation, respectively. Complementing this overview, Figure 1 illustrates the feedback interconnection among gut, immune, and endocrine pathways driving “metaflammation”, contributing to the progression of various metabolic disorders.

### 1.2. The Need for Integrated Approaches

The traditional view that obesity can be solved simply by “moving more and eating less” has been increasingly challenged as an oversimplification of a highly complex disease [110]. Studies have shown that powerful homeostatic mechanisms in individuals with obesity can hinder weight loss and promote further weight gain, making short-term behavioural or medical interventions often insufficient for long-term success [4]. The enormous complexity of causal factors and their interrelationships in the development of obesity means that current weight-loss strategies targeting only the individual may not address the most significant underlying causes of energy imbalance [4]. Therefore, recent research emphasises the need for a holistic understanding that integrates molecular, neuroendocrine, immunometabolic, and epigenetic mechanisms is essential for designing durable, personalised, and systemic therapeutic approaches [72,89,110].

Obesity is increasingly recognised not merely as a consequence of caloric imbalance, but as a complex, chronic, and relapsing systemic disease demanding comprehensive, integrated, and personalised therapeutic strategies. This paradigm change requires moving beyond conventional thinking about weight management to accommodate the complex interplay of neuroendocrine, immune, metabolic, and epigenetic dysfunctions, along with the preeminent influence of gut microbiota imbalances [73,90,92,110]. 

In this context, obesity has been increasingly recognised as a chronic, relapsing, and multifactorial disease, rather than a condition arising from personal failures, so that therapeutic interventions can be introduced to work with individuals. The newer conception emphasises the necessity for a multifaceted approach that targets inflammation, microbiota, neuroendocrine circuits, and immunometabolic cross-talk [111,112,113].

Epigenetic landscapes, through mechanisms such as DNA methylation, histone modifications, and microRNA regulation, have been identified to act as essential mediators in translating environmental exposures into changes in gene expression that can persist across generations [7,114,115]. Emerging evidence suggests that some of these changes could indeed be reversed with targeted lifestyle interventions, bariatric surgery, and emerging pharmacotherapies.

Effective management has been shown to require a multidisciplinary care, integrating expertise from various healthcare professionals to address the multifaceted nature of the disease [116,117]. Evidence indicates that such integrated approaches are crucial for sustained success, particularly in adolescents, where lifestyle modifications, including diet, physical activity, and behavioural therapy, form the cornerstone of intervention [117].

A significant advancement within this integrated approach is the rise of precision medicine, which tailors interventions to an individual’s unique genetic, environmental, and lifestyle characteristics [118]. Multiomics data, comprising genomic, epigenomic, proteomic, metabolomic, and microbiomic profiles, have been increasingly applied to gain a deeper understanding of specific subtypes of obesity and the underlying pathway mechanisms [110]. As such, the biomedical researchers agree that the biological insight gained through this approach could enable targeted nutrition and therapeutic measures to be designed to optimise outcomes and minimise adverse side effects [110,119].

Although communication between the healthcare and community sectors is crucial for paediatric obesity care and treatment, as well as the coordination of recommendations and responses, studies have highlighted that technology can play a significant role in enhancing healthcare-community communication, preventing barriers to collaboration, and fostering links that promote well-being and healthy weight status [120].

Thus, this review aims to emphasise that metaflammation is not just a mere secondary of obesity but the key mechanism underlying obesity-related pathology. By synthesising evidence on the roles of adipose tissue dysfunction, gut dysbiosis, bile acid signalling, neuroendocrine disruption, and systemic spillover, the review proposes a unified framework in which obesity is a disorder of chronic immunometabolic stress. Recognising metaflammation as a fundamental process defines obesity as an inflammatory disease that affects the entire organism, explaining its connections to diabetes, cardiovascular disease, neurodegeneration, and other conditions. This perspective emphasises that interventions should not only focus on weight reduction but also re-establish metabolic-immune homeostasis, whether through modulation of diet, microbiota, inflammatory pathways, or neuroendocrine signalling. Ultimately, the review seeks to provide a comprehensive synthesis for understanding obesity as a cycle of metaflammation, where energy overload and immune system activation become intertwined, leading to systemic dysfunction as an inevitable consequence.

## 2. Adipose Tissue: A Dynamic Immunometabolic Organ

### 2.1. From Energy Reservoir to Active Organ

The traditional view of adipose tissue as a simple energy store has been significantly revised in recent decades. Thus, recent studies have shown that adipose tissue is a lively and multifunctional organ with essential endocrine and immune functions that go well beyond storing lipids [111,121,122,123,124]. Research has shown that, under normal conditions, adipocytes help maintain overall body balance by releasing a variety of bioactive substances, including adipokines, cytokines, chemokines, and lipid mediators, which regulate metabolism, insulin action, and immune defence [124,125,126]. Therefore, adipose tissue is seen as an active endocrine and immune organ, with its dysfunction playing a key role in the development of chronic metabolic diseases. For instance, according to published data, adipokines, such as LEP and adiponectin, are key regulators of energy balance, glucose, and lipid metabolism. Leptin, for example, influences satiety and energy expenditure, while adiponectin enhances insulin sensitivity and contributes to metabolic fitness [127,128,129]. Several studies have also reported that the factors secreted by adipocytes directly influence the insulin response in various tissues, ensuring appropriate glucose uptake and utilisation, which is important for maintaining optimal systemic insulin sensitivity. Furthermore, research findings indicate that adipocytes participate in immune function by releasing cytokines and chemokines, thereby modulating inflammatory responses and influencing the recruitment and activity of immune cells within both adipose tissue and the broader physiological system. Collectively, these studies have led to the recognition of adipose tissue as an active endocrine and immune organ, with its dysfunction playing a key role in the development of chronic metabolic diseases.

### 2.2. Adipocyte Hypertrophy and Dysfunction

In the context of excessive adipose expansion induced by chronic caloric surplus, several studies have reported that the microenvironment of adipose tissue undergoes significant remodelling. Adipocyte hypertrophy, local hypoxia, and endoplasmic reticulum have been shown to stress trigger a molecular cascade that shifts the tissue towards a dysfunctional, pro-inflammatory phenotype [2,6]. Activated under these conditions, hypoxia-inducible factors (HIFs) promote the transcription of genes associated with inflammation, fibrosis, and metabolic impairment [130]. 

Previous research has demonstrated that dysfunctional adipose tissue, which is mainly characterised by hypertrophied adipocytes, upregulates inflammation, increases fibrosis, and impairs vascular function, being of great etiopathogenic relevance to obesity [131]. An increased accumulation of visceral adipose tissue contributes to chronic low-grade inflammation, which disrupts insulin receptor-mediated insulin signalling. From this point on, it was emphasised that the locally produced inflammatory cytokines such as TNF-α and IL-1β, secreted by adipose tissue macrophages (ATMs), further escalates and directly interferes with insulin action [31,41]. 

Furthermore, several studies have demonstrated that lipid species elevated by diet or obesity, including free fatty acids, stimulate inflammation by activating Toll-like receptors and thereby strengthen the downstream Nuclear Factor kappa-light-chain-enhancer of activated B cells (NF-kB) signalling cascade, which further upregulates the synthesis and secretion of chemokines, such as Ligand chemokine CC Motif 2 (CCL2) by adipocytes, thus increasing macrophage infiltration [31]. Researchers have shown that this ongoing infiltration results in an inflammatory transformation of adipose tissue, which is directly proportional to the severity of insulin resistance phenomenon [132]. Therefore, the persistent excess of calories specific to obesity has been reported to initiates a cascade of molecular events, including dysregulation of fatty acid homeostasis, adipocyte hypertrophy, and localised hypoxia, all of which converge to activate key inflammatory signalling pathways such as c-Jun N-terminal Kinase (JNK) and NF-kB, further perpetuating adipose tissue inflammation [41]. In turn, has been shown that this sustained inflammation impairs insulin signalling, contributing to systemic insulin resistance and the progression of metabolic disorders [6,133]. Moreover, it was observed that HIF-1α, activated in expanding adipose tissue, not only drives metabolic adaptations but also promotes inflammatory gene expression, linking cellular stress to immune activation [111,130].

### 2.3. Adipokines and Systemic Impact

Over the past decade, there has been a growing interest in understanding the systemic implications of adipokines in obesity. Thus, of particular significance is the dysregulation of the leptin/adiponectin axis. Research has consistently shown that LEP levels rise with adiposity, yet leptin resistance at the hypothalamic level attenuates its anorexigenic effects, favouring hyperphagia and weight gain [7,17]. Meanwhile, it was reported that adiponectin, a potent anti-inflammatory and insulin-sensitising adipokine, declines, exacerbating endothelial dysfunction and increasing cardiovascular risk [134]. Likewise, it was observed that the altered adipokine profile, particularly the reduced levels of anti-inflammatory adiponectin and elevated pro-inflammatory cytokines like TNF-α, further impairs insulin signalling and systemic glucose homeostasis [18].

### 2.4. Immune Cell Infiltration and Remodelling

One of the most prominent features of dysfunctional adipose tissue was shown to be the massive infiltration of immune cells, particularly M1-type macrophages, cytotoxic T lymphocytes, and NK cells, which creates a chronic low-grade inflammatory environment [1,5]. The immune shift is characterised by the tissue infiltration of classically activated macrophages, cytotoxic CD8+ T cells, Th1 and Th17 cells, neutrophils, and natural killer cells. Specifically, obese white adipose tissue was observed to exhibit a marked increase in pro-inflammatory immune cells such as M1-like macrophages, mast cells, dendritic cells, and CD8+ T lymphocytes, contrasting sharply with the anti-inflammatory milieu observed in lean white adipose tissue (WAT) [3]. This observation is sustained by other researchers who observed that, while lean adipose tissue maintains a Type 2 immune profile characterised by anti-inflammatory cytokines, such as IL-4 that sustain M2 macrophage polarisation, obesity shifts this balance towards a pro-inflammatory Type 1 response with phagocytic immune cell activity. These changes include a significant increase in the total number of immune cells within the adipose tissue, particularly macrophages [122].

Macrophage infiltration into adipose tissue has been identified to be a critical event in obesity, significantly contributing to metabolic dysfunction [135]. Several studies have shown that these ATMs are highly plastic, exhibiting phenotypic shifts to an anti-inflammatory M2-like phenotype in lean states. In contrast, in obesity, they adopt a pro-inflammatory M1-like phenotype that exacerbates both local and systemic inflammation [136]. Altered lipid metabolism in these macrophages promotes lysosomal dysfunction, amplifying inflammatory gene expression, which drives macrophage polarisation [137]. Evidence also suggests that the switch in macrophage phenotype is accompanied by an increased influx of monocytes that differentiate into M1-like macrophages within the tissue, thereby further amplifying the inflammatory cascade [3]. Furthermore, mucosal-associated invariant T cells have been shown to exacerbate insulin resistance and impede glucose and lipid metabolism by promoting inflammation in adipose tissue through M1 macrophage polarisation [138] and metabolic dysfunction [139]. This intricate cross-talk between adipocytes and ATMs was shown to further contribute to the metabolic dysfunction observed in obesity [140]. Beyond macrophages, other immune cell populations, including neutrophils, B cells, and specific subsets of T lymphocytes, also undergo significant phenotypic and functional changes within the adipose tissue of obese individuals, collectively contributing to the perpetuation of chronic inflammation and metabolic disturbances [121]. Activated M1 macrophages secrete TNF-α, IL-1β, and IL-6, thereby amplifying the recruitment of additional immune cells and perpetuating a positive feedback loop of inflammation [2,141]. Moreover, the formation of foam cells, lipid-laden macrophages within adipose tissue, has been reported to further contributes to tissue fibrosis, metabolic disruption, and local vascular rarefaction [3,137].

The chronic, low-grade inflammatory state within adipose tissue has been reported to not only impair local metabolic functions but also contribute to the systemic insulin resistance and dyslipidemia characteristic of obesity [142]. Concomitantly, the dysregulated trafficking of leukocytes into adipose tissue further exacerbates this pro-inflammatory shift, ultimately contributing to impaired insulin signalling and β-cell dysfunction, which can lead to hyperglycaemia [143]. The interplay between immune cells and adipocytes within this inflamed adipose tissue microenvironment has been described to create a vicious cycle that underpins the pathogenesis of obesity-associated metabolic disorders. Evidence from experimental and clinical studies indicates that an intricate immunological remodelling within the adipose tissue is a major component of systemic metabolic complications, thereby establishing a direct link between chronic inflammation and insulin resistance, as well as T2DM. Since ATMs have been identified to play a leading role in the inflammatory cascade, researchers concluded that their activation and phenotypic transition are major determinants of obesity-induced metabolic dysfunction [114]. For instance, M1 macrophages secrete significant amounts of pro-inflammatory cytokines and reactive oxygen species (ROS); conversely, M2 macrophages confer anti-inflammatory responses and are involved in tissue repair [144].

The metabolic reprogramming in macrophages was shown to increase glycolysis, as well as to support the secretory and phagocytic activities of these cells by providing rapid ATP production and the necessary substrates for anabolic pathways that generate proteins and ROS [130]. In M1-polarised ATMs, an elevated glycolytic flux metabolic phenotype is induced largely by HIF-1α activation, which in turn induces the secretion of IL-1β, a pro-inflammatory cytokine [130]. It was observed that this metabolic switch is a distinguishing feature of the chronic, low-grade inflammation observed in obesity, as opposed to an acute inflammatory reaction. Through an interplay between obesity and chronic inflammation, the trained immunity of macrophages sustains metabolic inflammation and insulin resistance even after the obese state has been reversed [145]. The sustained epigenetic reprogramming poses a daunting challenge for resolving obesity-associated metabolic disruptions, and, as other researchers propose, the approach should encompass addressing both short-term inflammatory reactions and long-term immunological memory [146].

Previous studies have demonstrated that the intricate cross-talk between adipocytes and tissue-resident macrophages mediates the inflammatory microenvironment, with adipocytes being able to secrete chemokines for macrophage recruitment and activation, thus entrenching the pro-inflammatory cascade [41,147]. This interplay is recognized as a key driver of metaflammation, as ATMs actively contribute to systemic low-grade inflammation in obesity through the secretion of pro-inflammatory cytokines such as TNF-α, IL-6, and IL-1β, which are major instigators of the insulin resistance phenomenon [148,149]. Evidence also indicates that this inflammatory environment in adipose tissues negatively affects the insulin signalling pathway through the mechanism of serine phosphorylation of insulin receptor substrate-1 (IRS-1), thereby impairing the response to insulin in target tissues [150].

Interference in insulin signalling has been reported to directly link inflammation in persistent adipose tissue to the manifestation of systemic metabolic disorders in the form of T2DM [151]. Arguably, numerous studies indicate that one of the primary pathophysiological occurrences of obesity-associated metabolic disorders is the infiltration of macrophages and lymphocytes into adipose tissue. Adiponectin likely influences this process by promoting M2-like macrophage polarisation [152,153]. Furthermore, researchers have shown that disruption of macrophage function in obese adipose tissue involves more than mere M1/M2 polarisation. It includes lipid metabolism dysregulation within macrophages, which further contributes to inflammation and insulin resistance [31]. Collectively, these findings support the view that this complex pathological cycle basically describes macrophage dysfunction affecting adipose tissue homeostasis, positioning this dysfunction at the centre stage for systemic insulin resistance in obesity [154].

Several studies have demonstrated that the chronic inflammatory state induced by macrophage infiltration and activation within adipose tissue directly impairs insulin signalling pathways in adipocytes and other peripheral tissues, thereby contributing significantly to systemic insulin resistance and the progression to T2DM [39,155]. This persistent inflammatory environment has also been reported to promote oxidative stress and alter endocrine variables, further disrupting insulin signalling pathways and contributing to glucose intolerance [133]. This intricate interplay between immune cells and adipocytes has been described to further exacerbate metabolic dysfunction, leading to a vicious cycle in which obesity drives inflammation, which in turn fuels insulin resistance [156]. This chronic inflammation, mainly induced by immune cells, will antagonise insulin sensitivity and, therefore, lead to the pathogenesis of metabolic disorders such as T2DM [157,158].

The activation of signalling pathways, such as NF-kB and JNK, has been reported to be regulated by obesity within the adipose tissue, and the released inflammatory factors affect the insulin signalling pathway, thereby leading to worsened insulin resistance [136]. According to recent studies, the metabolic state of adipocytes themselves can directly mediate the inflammatory response, suggesting that adipocyte dysfunction may precede and even trigger macrophage recruitment and activation [137].

However, inflammation is not the sole cause of obesity [137]. It is worth noting that while the exact initiators of obesity-induced adipose tissue inflammation remain to be fully delineated, disrupted fatty acid homeostasis, enlarged adipocyte size and death, local hypoxia, mitochondrial dysfunction, and endoplasmic reticulum stress have all been implicated as the mechanistic intermediates linking caloric excess to chronic inflammation [41].

**Figure 2:**
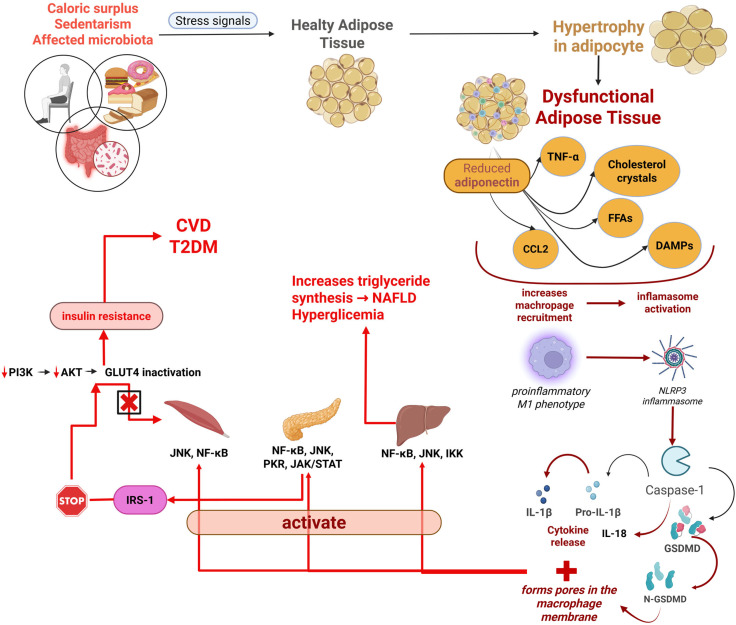
Molecular and cellular mechanisms linking hypertrophic adipocytes to systemic metaflammation and insulin resistance. Enlarged adipocytes release free fatty acids (FFAs), cholesterol crystals, danger-associated molecular patterns (DAMPs), and pro-inflammatory cytokines/chemokines such as TNF-α and MCP-1. These mediators recruit macrophages, which polarise toward a pro-inflammatory M1 phenotype. Within both adipocytes and macrophages, the activation of the NLRP3 inflammasome and caspase-1 leads to the secretion of IL-1β and IL-18, thereby amplifying local and systemic inflammation in a vicious cycle. The released cytokines activate downstream intracellular signalling cascades in metabolic tissues (liver, skeletal muscle, pancreas), including NF-kB, JNK, IKK, PKR, JAK/STAT, which impair insulin receptor substrate-1 (IRS-1) activity, reduce AKT/PI3K pathway signalling, and block GLUT4 translocation. The cumulative effect results in insulin resistance, hyperglycaemia, and dyslipidemia, promoting progression toward type 2 diabetes mellitus (T2DM), non-alcoholic fatty liver disease (NAFLD), and cardiovascular disease (CVD).

A representative diagram illustrating the molecular and cellular mechanisms linking hypertrophic adipocytes to systemic metaflammation and insulin resistance is included in Figure 2.

## 3. Metaflammation: The Core Inflammatory Driver of Obesity

### 3.1. Definition and Characteristics

Metaflammation has been defined in the literature as a chronic low-grade systemic inflammation associated with an inflammatory state induced by nutritional overload of metabolic cells [159]. This chronic inflammation is the essential link between obesity and many metabolic and cardiovascular complications. Thus, the proinflammatory state was shown to contribute to T2DM by increasing peripheral insulin resistance and impairing insulin secretion [160]. Metaflammation, unlike classic inflammation, is chronic but of low intensity and is associated with metabolic deregulation rather than invasion of a pathogen [160]. Research has further demonstrated that metaflammation involves a complex interplay between nutrient excess and immune responses, leading to cellular dysfunction and systemic pathology [159]. Hence, this process drastically contributes to significant global public health challenges presented by obesity and its co-morbid formations, including T2DM and cardiometabolic diseases [159]. A complex interaction between immune cells and metabolic tissues characterises metaflammation. Macrophages, notably in adipose tissue, were observed to undergo phenotypic alteration, thereby maintaining a cycle of inflammation and promoting the development of insulin resistance [161]. This sustained inflammatory environment subsequently was reported to impair cellular responses to insulin, favouring a state of systemic insulin resistance intricately associated with dyslipidemia and hyperglycaemia [161].

### 3.2. Cellular and Molecular Triggers

Obesity-induced inflammation has been reported to involve multiple organs: adipose tissue, pancreas, liver, skeletal muscle, heart, and brain [162], with a particular focus on the impact of weight cycling, the gut microbiome, adipose tissue signalling, and immune cell cross-talk, as the key contributors [91]. One widely accepted mechanism is adipose tissue dysfunction, where enlarged or hypertrophied adipocytes, which occur in obesity, become stressed. This stress was incriminated to cause them to release pro-inflammatory molecules, such as cytokines and chemokines [3,93,114,163]. During the next step, immune cells release substances that were presented to act as signals to recruit immune cells, particularly macrophages, into the adipose tissue [3,126]. Once recruited, these immune cells contribute to and amplify the existing inflammation, creating a self-perpetuating cycle [3].

Furthermore, metaflammation is thought to originate from metabolic cells in response to excess nutrients and energy, thereby playing a pivotal role in the systemic expansion of metabolic diseases such as T2DM [46,159]. The persistent activation of pro-inflammatory signals by immune cells has been reported to further exacerbates this unresolved inflammatory mechanism, contributing to impaired glucose uptake and insulin action through the recruitment of inflammatory kinases, such as JNK, KK, and protein kinase R (PKR), in metabolic tissues [45]. Intracellular inflammatory pathways, such as NF-kB, Janus kinases (JAK)/Signal Transducers and Activators of Transcription (STAT), and the activation of the NLRP3 inflammasome, orchestrate metaflammation, which contributes to the pathogenesis of insulin resistance, T2DM, and cardiovascular disease [111].

The NLRP3 inflammasome has been recognised as a critical sensor of metabolic stress, primarily activated in dysfunctional adipose tissue, and thus significantly contributes to the systemic inflammatory response observed in obesity [34,164,165]. As a component of the innate immune system, the NLRP3 is activated by pathogen-associated molecular patterns and danger-associated molecular patterns, leading to the processing and secretion of potent pro-inflammatory cytokines, such as IL-1β and IL-18 [32,34,39,41]. This activation by endogenous danger signals, such as palmitic acid and cholesterol crystals, suggests that NLRP3 activation serves as the catalyst for chronic metabolic diseases characterised by continuous inflammation [159].

In the context of obesity, the NLRP3 was presented to act on cellular stress signals released from stressed or dying adipocytes in the form of danger-associated molecular patterns (DAMPs), which strongly activate the inflammasome [31]. Specifically, the released fatty acids induce NLRP3-ASC inflammasome activation, thereby interfering with insulin signalling and reducing insulin sensitivity [166]. Furthermore, in metabolic tissue, NLRP3 activation continues to sustain insulin resistance and cellular dysfunction, thereby accelerating the progression of obesity-related comorbidities [45].

In Table 2 below, we present a more comprehensive overview of how various factors trigger the NLRP3 inflammasome and the far-reaching metabolic consequences in the context of the reported literature on obesity. The experimental models presented in this table are either preclinical or clinical. However, it is noteworthy to mention that current recommendations are to differentiate between the preclinical forms of obesity characterised by excess adiposity without organ dysfunction, and the clinical ones dominated by excess deposition of adipose tissue accompanied by metabolic consequences, organ dysfunction, and related issues.

Inflammasome activation alone has been reported to be enough to cause inflammation in obese adipose tissue; other mechanisms are triggered by the hypoxic conditions and mechanical stress imposed on adipose tissue as it expands [31]. Specifically, dietary lipids, particularly free fatty acids, have been shown to directly stimulate inflammatory pathways by binding to Toll-like receptors, such as TLR2 and TLR4. Subsequently, the complex activates NF-kB signalling and enhances the synthesis of chemokines, including CCL2, in adipocytes. Once activated, NF-kB can increase the synthesis and secretion of chemokines, such as CCL2, by adipocytes, thereby facilitating the recruitment of immune cells. This activation, in turn, exacerbates adipose tissue inflammation and contributes to systemic insulin resistance [31].

Moreover, the recognition of both endogenous and exogenous ligands by pattern recognition receptors, such as Toll-like receptors, has been shown to initiate or promote chronic inflammation in obesity by activating downstream NF-kB signalling, which in turn upregulates the synthesis of chemokines like CCL2 [31,238,239]. This inflammatory cascade, characterised by the persistent secretion of pro-inflammatory cytokines such as TNF-α and IL-1β from ATMs, directly impairs insulin signalling pathways [31]. Such sustained activation contributes to the development of systemic insulin resistance, thereby exacerbating the metabolic dysfunction characteristic of obesity [158].

In addition to TLR signalling, nucleotide-binding oligomerisation domain-containing protein 1 (NOD1) has been identified as another key protein that is involved in the inflammatory response, whose signalling in various insulin-sensitive metabolic tissues progressively increases in high-fat diet-fed mice, correlating with the progression of insulin resistance [141,151]. This upregulation of NOD1, particularly in skeletal muscle, adipose tissue, and the liver, has been associated with an intensified inflammatory response and was causally linked to both peripheral and hepatic insulin resistance [151,240]. Moreover, activation of NOD1 in hematopoietic cells can further promote changes in macrophage polarisation, shifting them towards a proinflammatory state, thereby amplifying systemic inflammation and insulin resistance [240]. Specifically, it has been shown that the activation of NOD1 reduces insulin-induced glucose uptake, indicating a direct link between innate immunity and insulin resistance in human adipocytes [241].

Beyond peripheral metaflammation, evidence has emerged that obesity-related inflammation extends to the central nervous system. By experimental studies, it was observed that this neuroinflammation can disrupt neuronal circuits that regulate appetite and metabolic energy, thereby promoting a vicious cycle of weight gain and further systemic dysfunction [28]. Such a complex interaction between peripheral metaflammation and central neuroinflammation highlights the profound impact of obesity on brain function and metabolic control [93]. Moreover, accumulating evidence suggests that hypothalamic inflammation, even before significant weight gain, can rapidly impair energy balance and contribute to obesity-associated insulin resistance by activating key inflammatory mediators, such as JNK and IkB kinase [242]. This microinflammation in the hypothalamus, often referred to as hypothalamic microinflammation, develops early in over-nutritional states due to the activation of pro-inflammatory signalling pathways, including NF-kB [243]. This rapid onset suggests that hypothalamic inflammation may be a cause rather than a consequence of obesity, initiating a cascade that disrupts the neuroendocrine regulation of energy homeostasis [93]. As so, the researchers conclude that the central nervous system, particularly the hypothalamus, plays a crucial role in maintaining glucose homeostasis by sensing changes in blood glucose levels and responding to glucoregulatory hormones, such as insulin and leptin [244].

### 3.3. Systemic Spillover and Organ-Specific Impact

This nuanced understanding highlights the systemic implications of adipose tissue dysfunction, particularly its role in spreading inflammation to distant organs and contributing to metabolic dysregulation [245]. The researchers reported that untreated inflammation leads to a phenomenon known as “systemic spillover”, in which chronic low-grade inflammation, originating primarily from dysfunctional adipose tissue, spreads beyond its original site and affects distant tissues and organs throughout the body. Circulating inflammatory factors, such as C-Reactive Protein (CRP), IL-1β, IL-6, CCL2, and TNF-α, are released into the bloodstream. Along with decreased levels of the anti-inflammatory factor IL-10, these factors can lead to endothelial injury that can impair vascular function, lead to hepatic steatosis, and myocardial remodelling [138,246]. This systemic dissemination of inflammatory mediators highlights the complex interplay between localised adipose tissue dysfunction and widespread metabolic pathology, leading to a state of chronic, low-grade inflammation that profoundly affects whole-body insulin sensitivity and glucose homeostasis [41,43,247]. Subsequently, this generalised inflammatory response has been shown to initiate a cascade of adverse effects on cellular function and organ systems, leading to an increased risk of chronic metabolic diseases [43,162]. Hypothalamic inflammation can develop even before significant weight gain with high-fat diet consumption, impacting energy balance and fostering overeating [5]. In the end, other organs and systems will be affected, leading to various metabolic complications [5,43]. 

Chronic low-grade inflammation extends beyond traditional metabolic tissues. In the central nervous system, circulating cytokines cross the blood–brain barrier, inducing microglial activation and neuroinflammation, processes that have been shown to be implicated in cognitive decline and increased risk of neurodegenerative diseases in obese individuals [5,126]. Studies have demonstrated that chronic inflammatory burden also contributes to disruption of central neuroendocrine pathways, affecting appetite regulation, mood, and energy expenditure [5,111]. Hypothalamic inflammation can occur with the consumption of a high-fat diet, even before significant weight gain, thus potentially affecting energy balance and promoting overeating [5]. As a result, other organs and systems could be affected, leading to various metabolic complications.

In the reproductive system, inflammation has been reported to interfere with gonadal steroidogenesis, impair gametogenesis, and alter the hypothalamic-pituitary-gonadal axis, leading to reduced fertility and syndromes such as polycystic ovary syndrome. As such, the researchers concluded that low-grade chronic inflammation represents the fundamental mechanistic link between obesity and its myriad complications, emphasising the need for anti-inflammatory strategies as integral components of obesity management [3,12,115].

Collectively, these findings indicate that metaflammation is not simply a secondary phenomenon, but a central pathological axis linking obesity to insulin resistance, T2DM, CVD, hepatic steatosis, cognitive impairment, and reproductive dysfunction. Therefore, we consider that, in the current context of the obesity epidemic, interventions must be personalised and multidisciplinary, and the approach to low-grade inflammatory processes should be initiated from the early stages of diagnosis.

## 4. Gut Microbiota Dysbiosis: A Key Modulator of Metabolic Dysfunction in Obesity

### 4.1. Dysbiosis and Metabolic Endotoxemia

Recent research has increasingly highlighted the role of gut microbiota as a pivotal modulator of host metabolism, immune responses and even behaviour. In obesity, a significant dysbiosis of the gut microbial ecosystem has been observed, characterised by reduced diversity, altered *Firmicutes*/*Bacteroidetes* ratios, and an increased capacity to obtain energy from the diet. The occurrence of dysbiosis has been reported to exacerbate the pathology, characterised by metabolic abnormalities resulting from a disturbed *Firmicutes*/*Bacteroidetes* ratio, accompanied by a decrease in the formation of SCFAs and an increase in systemic endotoxemia, which triggers a widespread immunometabolic dysregulation [11,13]. Specifically, in obese individuals, an increased *Firmicutes*/*Bacteroidetes* ratio has been observed to facilitate the breakdown of complex carbohydrates into absorbable SCFAs such as acetate, propionate, and butyrate. Although SCFAs typically exert beneficial anti-inflammatory effects, in the context of dysbiosis, their overproduction contributes to excessive lipid storage and insulin resistance. In addition to altered energy metabolism, intestinal dysbiosis promotes increased intestinal permeability, often referred to as “leaky gut”, allowing bacterial lipopolysaccharides to enter the circulation, which triggers low-grade systemic inflammation and contributes to the development of obesity and diabetes [71]. This chronic low-grade systemic inflammation is initiated by activation of TLR4 in adipocytes [5,11,31] and has been shown to lead to the development of “metabolic endotoxemia”, which triggers systemic inflammation and insulin resistance [103,248]. This process activates proinflammatory pathways and increases oxidative stress [91]. Ultimately, this activation further triggers downstream signalling pathways that culminate in the secretion of proinflammatory cytokines, thereby intensifying the systemic inflammatory state and contributing to insulin resistance [91,249]. Collectively, these findings suggest that dysbiosis plays a dual role: it acts both as a consequence of metabolic imbalance and as an amplifier of inflammation, oxidative stress, and insulin resistance.

Although dysbiosis has been consistently associated with obesity and metabolic syndrome, the available scientific data does not support the view that gut microbiota imbalance is the primary driver of obesity. Instead, it represents an important component of a multifactorial network involving diet, genetics, neuroendocrine regulation, and chronic metaflammation. In this context, gut dysbiosis acts as both a consequence and an amplifier of metabolic dysfunction, contributing to systemic inflammation, insulin resistance, and altered energy homeostasis.

### 4.2. Microbiota-Bile Acid Axis

The gut microbiota has been recognised to play a crucial role in the metabolic transformation of bile acids, forming a critical “gut-liver-bile acid axis” that significantly influences host metabolism and contributes to the development of obesity [101,102]. Upon reaching the intestine, primary bile acids are converted to secondary bile acids by the gut microbiota [250,251]. This biotransformation is a critical step that fundamentally alters the signalling properties and metabolic functions of bile acids [252]. For example, many gut bacteria, particularly those of the phylum Bacteroidetes, possess bile salt hydrolase (BSH) activity. These enzymes, which deconjugate primary bile acids, have been reported to influence the overall bile acid pool and affect host lipid metabolism, cholesterol homeostasis, and even weight gain [250,253,254,255].

Other microbial enzymes, such as those involved in 7α-dehydroxylation, further convert primary bile acids into secondary bile acids, deoxycholic acid and lithocholic acid [250]. Then, these primary and secondary forms act as a complex network of signalling molecules. These modified bile acids exert their profound effects on host metabolism primarily through the activation of specific receptors, particularly the nuclear FXR and TGR5 [256,257,258,259]. Both receptors are expressed in diverse metabolic tissues, including liver, intestine, and adipose tissue, and their activation regulates critical processes such as glucose and lipid metabolism, energy expenditure, and inflammatory responses [260,261,262,263]. For instance, FXR activation can suppress bile acid synthesis in the liver and influence glucose homeostasis, whereas TGR5 activation is linked to increased energy expenditure and improved glucose tolerance [256,257].

In the context of obesity, the occurrence of dysbiosis and alteration of the gut microbiota, such as changes in the *Firmicutes*/*Bacteroidetes* ratio [102], was often observed to lead to a dysregulation of bile acid storage. This dysbiosis, characterised by changes in microbial composition and microbial enzymatic activity, can significantly alter the amount and composition of primary and secondary bile acids [101,103,264]. Such changes can affect host metabolism, contributing to an increased risk of obesity by affecting energy balance, insulin sensitivity, and systemic inflammation, as a result of modulating the expression of FXR, TGR5, and other signalling pathways [101,103,264]. This highlights how dysregulation of the microbiota-bile acid axis profoundly contributes to the development and progression of metabolic disorders. A summary of findings from the current literature describing the specific roles of different microbial taxa in bile acid transformation and their metabolic consequences is provided in Table 3.

### 4.3. Microbiota and Gut Hormones Modulation

Recent studies have highlighted the impact of gut microbiota on the endocrine axis. Dysbiosis can modulate circulating levels of ghrelin, GLP-1, and PYY by producing SCFAs, which stimulate their release from L-cells in the ileum and colon [13,295]. Through its metabolites, the gut microbiome influences ghrelin signalling [296]. For example, elevated blood levels of acetate, a short-chain fatty acid produced by certain gut bacteria, have been shown to increase ghrelin production in the stomach [297]. This suggests that the types and amounts of acids SCFAs produced by the microbiota may directly influence ghrelin secretion. Regarding GLP-1 secretion, SCFAs produced by intestinal bacterial fermentation of dietary fibers, especially butyrate, stimulate enteroendocrine L cells to release GLP-1, thereby contributing to improved glucose homeostasis and satiety [298,299,300,301]. The mechanisms are triggered by the activation of specific G-protein-coupled receptors, such as Free fatty acid receptors (FFAR2 and FFAR3), which are expressed on enteroendocrine L cells [299,302,303]. Activation of these receptors by SCFAs leads to an increase in intracellular calcium levels and the release of GLP-1 [299]. Through this mechanism, GLP-1 was considered to play an essential role in orchestrating the interaction between the gut, microbiota and the immune system to maintain intestinal barrier integrity, inflammation and metabolic homeostasis [304]. On the other hand, the gut microbiota was shown to influence circulating PYY levels by activating G-protein-coupled receptors through SCFAs, resulting from the fermentation of dietary fibers [90,302]. According to Farzi’s research, there is a bidirectional relationship between PYY signalling and the microbiota, as the lack of PYY signalling has been shown to lead to distinct changes in the composition of the gut microbiome in experiments conducted in animal models [305]. A similar bidirectional relationship has been observed for other gut hormones [306]. Other gut hormones, including cholecystokinin, gastric inhibitory polypeptide, and serotonin i.e., 5-hydroxytryptamine (5-HT)), are also regulated by microbial metabolites, influencing metabolic processes such as glucose metabolism, insulin sensitivity, and adiposity [306]. The mechanism by which the gut microbiota modulates the secretion of these hormones involves complex interactions with enteroendocrine cells, often mediated by SCFAs and other microbial metabolites [307]. For instance, microbial metabolites like indole, a tryptophan derivative, can modulate GLP-1 and satiety, while dietary fat can indirectly influence gut microbiota composition, leading to dysbiosis and a pro-inflammatory state [90].

This complex interaction highlights how microbial metabolites, especially SCFAs derived from the fermentation of dietary fibers, and others, directly influence host metabolism and endocrine function by modulating the release of gut hormones such as GLP-1 and PYY. In addition, microbial metabolites, including SCFAs and secondary bile acids, influence hormonal pathways through epigenetic modifications and direct receptor-mediated mechanisms [90,113,306].

## 5. Neuroendocrine Dysregulation and Obesity

### 5.1. Hypothalamic Inflammation

Recent research highlights that obesity is not just a peripheral metabolic disorder, but a condition deeply rooted in central nervous system dysfunction. As mentioned above, the hypothalamus, a key brain region involved in the regulation of energy homeostasis, undergoes profound inflammatory changes in response to excessive nutritional intake and adiposity, thereby disrupting the delicate control of hunger, satiety, and energy expenditure [5,111]. One of the earliest and most critical alterations observed in obesity is hypothalamic inflammation, characterised by microglial activation, astrogliosis, and the secretion of proinflammatory cytokines such as TNF-α and IL-6 [5]. This neuroinflammation, triggered by nutrient excess and lipid overload, has been shown to lead to impaired signalling of anorexigenic hormones such as LEP and insulin, fostering a state of central LEP and insulin resistance. The central resistance further aggravates weight gain by interfering with normal satiety cues and encouraging increased food consumption [308]. Neuroimaging studies using quantitative magnetic resonance imaging (IMRI) have provided additional evidence of these alterations, revealing increased water content and gliosis within hypothalamic regions, consistent with the suggestion of ongoing neuroinflammatory processes [309].

### 5.2. Leptin and Insulin Resistance

Although both insulin and leptin are crucial afferent signals to the central nervous system that reduce food intake, LEP has been reported to play a more important role in the hypothalamic control of energy homeostasis [114]. Leptin, a major adipokine produced by white adipose tissue, plays a key role in the hypothalamic regulation of appetite and energy expenditure [7,17,310]. In the context of obesity, chronic hyperleptinemia has been shown to induce paradoxical LEP resistance, attenuating hypothalamic anorexigenic responses and leading to persistent hyperphagia and weight gain [7,17]. This central desensitisation has been linked to alterations in LEP receptor signalling pathways, such as JAK2-STAT3 and Extracellular signal-regulated kinase (ERK), as well as increased inhibitory activity of the regulatory protein named Suppressor of cytokine signalling (SOCS)3 [5]. The existence of elevated LEP levels in obese individuals has been interpreted as evidence that some cases of human obesity may arise as a result of reduced LEP action in the brain, rendering individuals unresponsive to pharmacological LEP treatment [114]. This resistance was often interpreted as a consequence of impaired LEP transport across the blood–brain barrier or defective intracellular signalling pathways in hypothalamic neurons [114].

Similarly, insulin, secreted by pancreatic β cells, acts on hypothalamic neurons to regulate glucose metabolism and satiety; however, it was often observed that obesity led to hypothalamic insulin resistance, characterised by impaired insulin signalling and impaired glucose uptake in the brain [311]. This central desensitisation contributes significantly to energy imbalance, perpetuating the cycle of increased food intake and weight gain [114].

This complex interaction suggests that, although both hormones follow specific common pathways in regulating appetite and energy metabolism, the role of LEP in controlling energy homeostasis by the brain appears to be more critical [19]. This observation is supported by research indicating that LEP deficiency leads to severe obesity, characterised by persistent hyperphagia, even in the presence of elevated insulin levels. In contrast, it was observed that insulin deficiency does not induce obesity [114]. Conversely, hyperleptinemia, commonly observed in obese individuals, has been associated with increased insulin resistance, suggesting a complex, bidirectional relationship between these two hormones in the context of metabolic dysfunction [312]. The existing disruptions in serotonin signalling compound this complex hormonal dysregulation. Particularly, disruption of the 5-hydroxytryptamine 2C (5-HT_2_C) receptor pathway has been reported to induce leptin-independent hyperphagia, thereby exacerbating both obesity and insulin resistance [313].

### 5.3. Hormonal Imbalance

Obesity profoundly disrupts hormonal homeostasis, affecting both peripheral and central endocrine axes, with significant consequences for metabolism and reproduction. Beyond LEP and insulin, a broader spectrum of hormonal imbalances, including changes in adipokines, gut hormones, and neuroendocrine regulators, has been implicated in the systemic dysfunction characteristic of obesity.

Ghrelin, the only known circulating orexigenic hormone, has been reported to display altered secretion patterns in obesity. Thus, despite increased fat mass, levels of active acyl-ghrelin are significantly reduced, disrupting normal modulation of meal initiation and energy storage [15]. Recent findings suggest that altered ratios of acyl-ghrelin to des-acyl-ghrelin contribute to the disruption of central control of appetite and metabolic processes [16,314].

In obesity, stress-related pathways, particularly the hypothalamic–pituitary–adrenal (HPA) axis, have also been shown to be chronically activated. Likewise, persistently elevated cortisol levels were shown to exacerbate central fat accumulation, promote insulin resistance, and perpetuate a vicious cycle of metabolic and neuroendocrine dysfunction [1,12].

Among the most affected pathways, the hypothalamic-pituitary-gonadal (HPG) axis was reported to become highly sensitive to metabolic and inflammatory changes induced by excess adiposity. This dysregulation is mediated by altered levels of adipokines, such as LEP and adiponectin, as well as by low-grade systemic inflammation, both of which directly interfere with normal ovarian function and hypothalamic pituitary signalling. Specifically, increased macrophage infiltration in the ovaries, mediated by CCL2 pathways, has been shown to directly impair ovarian function. Moreover, the elevated levels of advanced glycation end-products (AGEs) in the serum and tissues of obese women may also exacerbate reproductive dysfunction [295]. Elevated LEP levels lead to LEP resistance, which impairs pulsatile secretion of gonadotropin-releasing hormone and ultimately impairs gonadotropin responsiveness [17,111]. This disruption was observed to be clinically manifested by anovulation in women and impaired spermatogenesis in men [315,316]. In addition, excessive aromatisation of androgens to oestrogens in adipose tissue has been shown to further exacerbate hypogonadism, especially in obese men. On the other hand, research on men indicated that elevated oestradiol levels inhibit the secretion of luteinising hormone (LH) and follicle-stimulating hormone (FSH), thus perpetuating reproductive dysfunction [14,317].

Sex hormone-binding globulin (SHBG) levels are also markedly reduced in obesity, limiting the bioavailability of androgens and oestrogens and amplifying endocrine imbalances [134]. Furthermore, it was observed that chronic low-grade inflammation associated with obesity is involved in the alteration of the functional capacity of Sertoli and Leydig cells in the testes and granulosa cells in the ovaries, further impairing fertility [18]. According to the literature, in young girls, early-onset obesity accelerates pubertal timing, mediated in part by leptin-driven activation of the HPG axis. In contrast, in boys, childhood obesity was linked to delayed pubertal development, likely due to a complex interplay between insulin resistance, altered adipokine profiles, and systemic inflammation [315].

Polycystic ovary syndrome (PCOS) is a classic example of endocrine disruption worsened by obesity. Hyperinsulinemia and systemic inflammation work together to promote hyperandrogenism, anovulation, and metabolic dysfunction in affected women [111]. It was shown that increased macrophage infiltration in the ovaries, mediated by CCL2 chemokine signalling pathways, directly impairs ovarian function [295]. Furthermore, the complex interaction between obesity and the hypothalamic–pituitary–ovarian (HPO) axis extends to the direct regulation of ovarian function, with adipokines such as chemerin, resistin, and visfatin directly influencing steroidogenesis and follicular development [295,318]. This complex hormonal environment, characterised by altered levels of gonadotropins, sex steroids, and adipokines, has been proven to critically affect delicate feedback mechanisms essential for proper reproductive function [295]. Taken together, in agreement with other researchers, we consider that obesity-induced hormonal dysfunctions represent a complex network of disrupted feedback loops, neuroendocrine rewiring, and inflammatory interference. Systemic inflammation, inherent in obesity, directly compromises ovarian folliculogenesis through immune cell infiltration and increased inflammatory markers in ovarian tissue, thus contributing to ovarian dysfunction [319].

### 5.4. Gut–Brain Axis

The enteric nervous system, an integral component of the gut–brain axis, is profoundly affected by obesity, as demonstrated by alterations in neuronal function and macrophage-neuronal interactions that collectively influence intestinal motility and nutrient absorption [91]. In parallel, the gut microbiota plays a crucial role in modulating nutrient absorption and energy expenditure, and the imbalances can lead to increased caloric intake and fat storage, thereby exacerbating obesity [11]. Recent research has identified a critical link between the gut microbiome and neuroinflammation that extends beyond the hypothalamus, suggesting that dysbiosis-induced factors transmit inflammatory signals to the brain, thereby contributing to neurodegeneration and ageing [320]. Researchers have observed that hippocampal inflammation occurs in obesity, which has been associated with cognitive impairment, memory deficits, and an increased risk of neurodegenerative diseases [62]. This intricate interplay involves constant bidirectional communication between the gut and brain, mediated by neural, endocrine, and immune pathways, that has been shown to influence systemic inflammation and overall metabolic health [302,321]. Elevated levels of circulating lipopolysaccharides resulting from gut dysbiosis were observed to cross the compromised blood–brain barrier, activating glial cells and perpetuating neuroinflammation, thus contributing to cognitive decline [322,323]. This chronic neuroinflammatory state, characterised by the activation of microglia and astrocytes, is further maintained by elevated levels of circulating fatty acids and LEP, which synergistically promote cytokine secretion through the TLR4/IKK/NF-kB pathway [5,91]. Additionally, it was observed that interactions between astrocytes and microglia in the brain contribute to central inflammation induced by obesity [91].

Furthermore, emerging studies that have aimed to analyse changes that occur at the level of the gut–brain axis suggest that microbial metabolites, such as SCFAs, and altered serotonergic signalling in the enteric nervous system can modulate central nervous system (CNS) inflammation and energy regulation [13]. The researchers observed that at the same time, the gut microbiota modulates satiety and eating behaviour through the gut–brain axis and influences the secretion of key neuromodulators, such as serotonin and dopamine. As such, it was postulated that the disruption of this signalling may lead to hedonic overeating and compulsive eating patterns observed in obesity. Beyond the direct impact on neuronal function, as mentioned above, it has been observed that obesity-induced gut dysbiosis promotes systemic inflammation, which can compromise the integrity of the blood–brain barrier, allowing peripheral inflammatory mediators to infiltrate the central nervous system and exacerbate neuroinflammatory processes [91]. This systemic inflammation, driven by metaflammation, can increase susceptibility to infections and alter immune responses [93].

Collectively, these findings emphasise that the complex interaction between gut microbiota, systemic inflammation, and the central nervous system is a multifaceted, bidirectional relationship that has a profound impact on both metabolic and neurological health [91,93]. This complex interaction involves constant bidirectional communication between the gut and brain, mediated by neural, endocrine, and immune pathways, which significantly influences systemic inflammation and overall metabolic health [320,321]. Within this framework, where microbial dysbiosis not only affects neurological function but also directly influences insulin sensitivity and cognitive performance, the gut–brain metabolic axis emerges as a crucial mediator [324]. 

**Figure 3:**
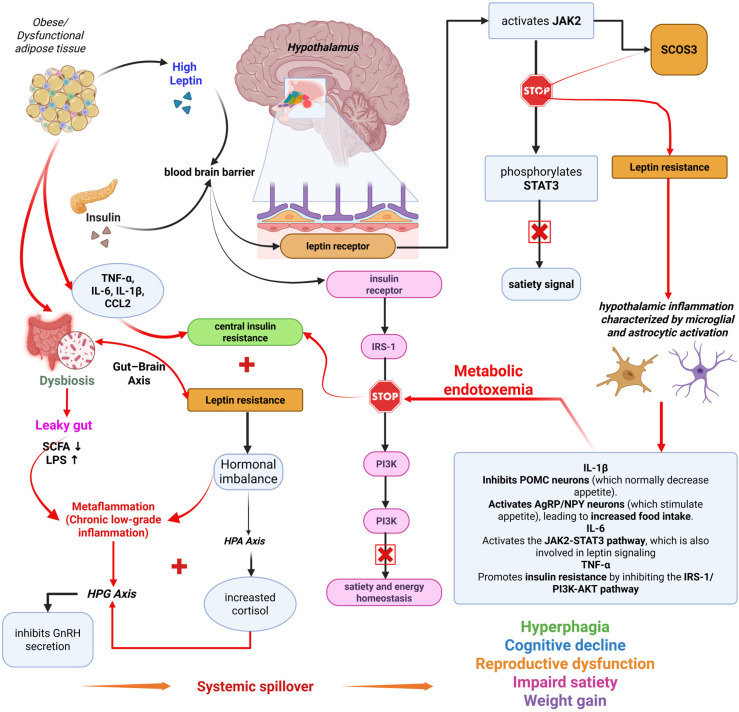
Neuroendocrine dysregulation in obesity: molecular, hormonal, and systemic mechanisms. Obesity is associated with hypothalamic inflammation characterised by microglial and astrocytic activation, resulting in impaired leptin and insulin signalling. Leptin resistance arises, despite elevated leptin secretion, from adipose tissue, due to reduced blood–brain barrier transport and intracellular inhibition of the JAK2–STAT3 pathway via SOCS3. Similarly, central insulin resistance develops through impaired IRS-1 and PI3K–AKT signalling, leading to defective energy homeostasis and satiety control. Hormonal imbalances further contribute to the decrease in active ghrelin, which blunts appetite regulation. At the same time, chronic activation of the hypothalamic–pituitary–adrenal (HPA) axis elevates cortisol, promoting central adiposity, systemic inflammation, and metabolic dysfunction. Disruption of the hypothalamic-pituitary-gonadal (HPG) axis results in hypogonadism, infertility, and abnormal pubertal development.

A representative diagram illustrating the complex interactions (molecular, hormonal, and systemic mechanisms) between gut microbiota, systemic inflammation, and the central nervous system in obesity is included in Figure 3.

## 6. Epigenetic Imprinting: Transgenerational Influences

### 6.1. Epigenetic Mechanisms in Obesity

Obesity is increasingly recognised not only as a metabolic disorder, but also as a condition profoundly shaped by epigenetic mechanisms that govern gene expression without altering the underlying DNA sequence. Epigenetic modifications, such as DNA methylation, histone modifications, and ncRNAs, particularly miRNAs, mediate the interaction between genetic predisposition and environmental exposures, serving as a molecular bridge between lifestyle factors and disease susceptibility [111,112]. 

In the last decade, researchers have suggested that obesity-induced epigenetic modifications, such as DNA methylation and histone acetylation, can be transmitted across generations, influencing the metabolic phenotype of offspring independent of genetic inheritance [12,72,73,82,87,206]. They demonstrate that obese individuals exhibit a distinct epigenetic signature, characterised by differential methylation of key genes involved in adipogenesis, lipid metabolism, insulin signalling, and inflammatory responses [325]. These include the PPARγ, LEP, and fat mass and obesity-associated (FTO) genes, whose epigenetic modifications correlate with increased adipocyte differentiation, insulin resistance and altered energy balance [18,111]. miRNAs also play a key role in mediating the epigenetic effects of obesity. The altered expression of miRNAs, such as miR-27a, miR-143, and miR-146a, which are present in adipose tissue and the circulation, contributes to insulin resistance, adipose tissue inflammation, and impaired lipid metabolism [31]. As a result, circulating miRNAs have been proposed as potential biomarkers for the early detection of obesity-induced metabolic dysfunctions [118]. An update on the dynamic nature of epigenetic modifications, their significant role in the development and progression of obesity, and the potential for intervention through lifestyle and therapeutic strategies, is highlighted in Table 4.

### 6.2. Early Metabolic Programming

In addition to lifestyle and nutritional habits, genetic and epigenetic factors significantly influence an individual’s susceptibility to obesity and their variable response to therapeutic interventions [7]. Increasing evidence indicates that the intrauterine environment has a profound impact on the developing foetal epigenome. Conditions such as maternal obesity, gestational diabetes, gestational inflammation, and excessive nutrient availability have been reported to predispose to obesity and metabolic disorders that occur later in life [8]. It has been observed that epigenetic changes during the foetal period, including DNA methylation patterns and histone acetylation, appear at the level of genes that regulate appetite, glucose metabolism, and adiposity, thereby predisposing the offspring to obesity and metabolic disorders that occur later in life [9]. This phenomenon, known as early metabolic programming, has been recognised to predispose children to an increased risk of obesity [7,115]. Additionally, recent studies have highlighted the paternal contribution to transgenerational epigenetic inheritance. Obesity in fathers has been associated with changes in sperm DNA methylation patterns, particularly in loci that regulate metabolic and inflammatory pathways, with a potential impact on embryonic development and metabolic outcomes in the next generation [312,388]. Collectively, these findings underscore that early-life environmental and nutritional exposures can induce persistent epigenetic alterations that modulate metabolic homeostasis across the lifespan.

### 6.3. Reversibility and Microbiota Influence

Emerging research suggests that some obesity-associated epigenetic changes are partially reversible, offering promising therapeutic avenues. It was observed that weight-loss interventions, whether through lifestyle modification, pharmacotherapy, or bariatric surgery, can lead to favourable remodelling of the epigenome, restoring the normal gene expression profiles in metabolic tissues [16,131,389]. For instance, significant improvements in the methylation of genes regulating insulin sensitivity and inflammatory responses have been observed following substantial weight loss [18,90].

Given the importance of SCFAs, recent studies have revealed that gut microbiota dysbiosis, frequently observed in individuals with obesity, may further influence the host epigenome through the production of metabolites, such as SCFAs, which modulate histone acetylation and DNA methylation [11,13]. It seems that this complex interplay underscores the ecological dimension of epigenetic regulation in obesity, emphasising the need for integrative therapeutic approaches that target both host and microbial factors. Like this, the complex interplay between host genetics, environmental factors, and the microbiome may create a dynamic epigenetic landscape that modulates an individual’s susceptibility to obesity and its associated comorbidities [82]. Such plasticity supports the concept that targeted interventions, ranging from dietary modulation and microbiota restoration to pharmacological epigenetic therapies acting on DNA methyltransferases or histone deacetylases, may partially reverse obesity-induced molecular signatures and restore metabolic homeostasis [73].

## 7. Therapeutic Strategies: Towards Integrated and Personalised Interventions

### 7.1. Targeting Metaflammation

Therapeutic strategies targeting metaflammation require multiple approaches. One strategy that has been proposed is related to personalised interventions, such as precision nutrition, which considers the individual’s genetic and epigenetic information, age, sex, and pathophysiological status. It is a novel approach used for the prevention and management of obesity-associated chronic diseases. High-throughput technologies, including metabolomics, genomics, transcriptomics, and gut microbiome analysis, have been proposed to be used in the development of personalised medicine for childhood obesity therapy [390]. Pharmacological agents, including novel anti-inflammatory compounds and gut microbiota modulators, are also being explored for their potential to ameliorate metaflammation and its metabolic consequences. However, the mechanisms by which excessive nutrient intake triggers oxidative stress in adipose tissue and the subsequent inflammatory response remain unclear [91]. 

Evidence from Kaushik and Anderson suggests that epigenetic changes, which can be dynamic and reversible with intensive lifestyle modifications, are associated with nutritional weight loss interventions and physical exercise [82]. Approaches such as anti-inflammatory pharmacotherapy, which includes inhibiting proinflammatory factors by blocking their release or modulating the inflammasome [391], have been observed to modulate inflammation in adipose tissue [392]. Addressing these multiple inflammatory pathways, especially the NLRP3 inflammasome, has been identified as a promising therapeutic strategy for alleviating metabolic complications associated with obesity [159,393]. 

Additionally, therapeutic strategies targeting metaflammation are crucial for mitigating obesity-induced cardiometabolic complications [160]. Over the last decade, therapeutic strategies aimed at restoring adipose tissue homeostasis by modulating inflammation, improving adipokine profiles, and adipose tissue plasticity have emerged and appear to be promising interventions for combating obesity-related complications [18,115,394].

### 7.2. Microbiota Modulation

The gut microbiome also offers potential intervention strategies for obesity and related diseases [30,395]. Dietary interventions, such as those rich in fermentable fibres and prebiotics, have been shown to beneficially modulate gut microbiota composition and function, thereby mitigating metaflammation and improving metabolic health [11,90,265,297,357,361]. Another strategy, involving the manipulation of gut microbiota through diet [94], prebiotics, or probiotics, has been reported to reduce intestinal low-grade inflammation and improve gut barrier integrity, thereby ameliorating metabolic balance and promoting weight loss [248]. Specific probiotic strains have been shown to be effective in reducing abdominal visceral fat [93]. Faecal microbiota transplantation, an innovative therapeutic strategy for modulating the microbiome, has shown great potential in treating obesity [93]. Furthermore, ongoing developments in technology and bioinformatics are enabling the creation of microbiome-manipulating capsules designed to promote a healthy, lean, and insulin-sensitive metabolic profile [73]. Finally, emerging pharmacological strategies are investigating the direct manipulation of microbial metabolites, such as SCFAs or bile acids, aiming to achieve metabolic benefits without necessarily altering the microbial community itself [13,113]. Since bile acids undergo microbial modification and exert critical effects on lipid and glucose metabolism, bile acid–targeted therapies are increasingly recognised as a potential route for metabolic intervention [93]. While the therapeutic modulation of the microbiota offers powerful expectation, interindividual variability remains a significant challenge. Personalised microbiota-based therapies, guided by metagenomic and metabolomic profiling, have been proposed as future directions of obesity management. However, rigorous, controlled studies are still needed to elucidate the precise mechanisms by which various microbial interventions affect host physiology and metabolism [396]. Further investigations into the complex interplay between specific microbial taxa and host metabolic pathways are crucial for developing highly targeted and effective microbiota-based interventions for obesity.

### 7.3. Precision Nutrition and Multi-Omics

Obesity is a complex and varied condition, with individuals responding differently to various interventions [118,119]. Precision nutrition, through the integration of multi-omics data, aims to address this by developing dietary strategies and interventions tailored to an individual’s specific characteristics, including genes, environment, lifestyle, and biological profiles [83,97]. This approach transcends traditional dietary guidelines by leveraging comprehensive biological insights to mitigate chronic low-grade inflammation.

Strategies utilising multi-omics data have been proposed to facilitate the identification of specific biomarkers and pathways involved in metaflammation, thus enabling targeted nutritional interventions that go beyond traditional dietary guidelines [119]. By integrating diverse datasets, including genomic, epigenomic, proteomic, metabolomic, and microbiomic information, and utilising various high-throughput technologies, researchers consider that they can provide a comprehensive understanding of an individual’s biology [119,397,398,399]. By characterising individuals at the molecular level, it was suggested that these omics assays will allow for a more refined classification of obesity into specific subtypes, or “phenotypes”, beyond conventional anthropometric metrics such as BMI, which then guide personalised nutritional and therapeutic approaches [119,390,400,401,402]. This stratification provides a foundation for more precise diagnosis, facilitating individualised preventive interventions and early treatments tailored to the specific characteristics of each type of obesity [390,400].

Genetic variation has been shown to influence both obesity susceptibility and individual response to interventions [403]. Genetic studies may help identify variants associated with obesity phenotypes, informing personalised nutrition and therapy [90].

Through metabolomics, small molecules in biological samples can be analysed to provide direct information about an individual’s current metabolic state and dynamic responses to interventions [404]. As such, metabolomic profiling can identify metabolic signatures and assess the efficacy of weight loss strategies, thereby contributing to more targeted and effective treatments [405].

This integrated approach allows for a deeper understanding of the molecular underpinnings of obesity and metaflammation, leading to more effective and individualised prevention and treatment strategies that enhance their efficacy and tolerability [406,407]. A comprehensive approach, including nutrigenomics, which investigates how genetic variations influence an individual’s response to nutrients, will facilitate the development of particular dietary recommendations [408].

The ultimate goal is to optimise treatment efficacy and minimise side effects by choosing the most appropriate strategy for each patient [119]. However, even though multi-omics offers significant prospects for personalised obesity management, challenges remain in translating these complex technologies into easily applicable and accessible clinical protocols [409]. Ongoing research is necessary to continue refining these approaches, paving the way for more effective and targeted interventions against the global obesity epidemic.

### 7.4. Targeting Macrophage Dynamics

Targeting the polarisation of ATMs toward an anti-inflammatory M2 phenotype represents another promising therapeutic path for attenuating obesity-associated metaflammation and improving metabolic outcomes [410]. Understanding the precise molecular mechanisms governing macrophage-adipocyte interactions may lead to the development of more effective interventions for obesity-related metabolic diseases [41,411].

One of the strategies developed by researchers relies on macrophages’ ability to eliminate adipocytes and lipid droplets, thereby maintaining tissue homeostasis [93]. Pharmacological interventions aimed at repolarising ATMs from M1 to M2 phenotypes, or at least attenuating M1-associated inflammatory actions, are significant for improving insulin sensitivity and ameliorating obesity-related metabolic complications [147,152]. Research indicates that strategies targeting metabolic pathways within macrophages are particularly promising for inducing a phenotypic shift in macrophages associated with obese adipose tissue, thereby shifting them toward a lean adipose tissue phenotype [412].

Interventions aimed at reprogramming macrophage metabolism have shown potential to shift macrophages within obese adipose tissue toward a lean-like phenotype, thereby reducing inflammatory tone and improving systemic metabolic outcomes [412]. Pharmacological inhibition of ceramide synthesis or activation of its degradation has been demonstrated to exert protective effects against systemic insulin resistance and metabolic inflammation [137,413,414]. Since excessive ceramide accumulation interferes with insulin signalling and promotes lipotoxicity, targeting ceramide pathways may provide a viable strategy for improving adipose function and reducing metaflammation [221].

Additionally, inhibition of key inflammatory kinases, including IκB kinase ε (IKKε) and TANK-binding kinase 1 (TBK1), using compounds such as amlexanox, has been shown to restore catecholamine sensitivity and reverse metabolic dysfunctions associated with obesity [31]. Collectively, these findings indicate that modulating macrophage dynamics and inflammatory signalling constitutes a promising therapeutic direction in the context of precision metabolic medicine.

A summary of key precision medicine initiatives focused on combating obesity by targeting underlying systemic dysfunctions is presented in Table 5.

Although recent advances in multi-omics, microbiome modulation, and precision medicine have expanded our mechanistic understanding of obesity, their translation into clinical practice remains limited. Current data are primarily descriptive and heterogeneous, lacking the large-scale, standardised evidence needed to guide clinical decision-making or patient-specific therapy. As a result, omics-based insights have improved disease stratification and biomarker discovery but have not yet provided reproducible clinical algorithms capable of guiding individualised treatment decisions. Future progress will depend on integrating molecular discoveries with large-scale clinical trials and harmonised data frameworks linking mechanistic knowledge to patient outcomes.

Moreover, it is important to recognise that excessive lipid accumulation within adipocytes remains the principal driver of metabolic dysfunction in obesity. Molecular, inflammatory, and epigenetic mechanisms amplify, but do not replace, this fundamental process. Therefore, emerging therapeutic paradigms should complement, rather than substitute, well-established interventions focused on energy balance, nutrition, and lifestyle modification. Closing this translational gap is essential for transferring all the experimental findings into clinically actionable strategies that can truly inform precision management of obesity

### 7.5. Extreme Obesity and Bariatric Surgery

Individuals with morbid obesity often experience a significantly reduced quality of life due to an increased prevalence of various obesity-related comorbidities, including psychological abnormalities, osteoarthritis, respiratory diseases, and gynaecologic issues [425]. Studies consistently report lower quality of life scores among individuals with severe obesity across multiple domains, including social acceptance, physical appearance, and physical functioning [426].

Globally, elevated body mass index (BMI) is a significant contributor to mortality and disability-adjusted life-years globally, with cardiovascular disease being the leading cause of death in obese persons [427].

According to Stein’s research [426], for adults, a direct BMI threshold (e.g., >40 kg/m^2^ or >35 kg/m^2^ with comorbidities) is used to define extreme obesity. However, for children and adolescents, the diagnosis of overweight and obesity, including extreme obesity, primarily relies on BMI percentiles relative to age and sex [426]. In their opinion, this approach accounts for the continuous changes in growth and body composition that occur throughout childhood and adolescence. For individuals aged 2 years and older, overweight is diagnosed if their BMI is between the 85th and <95th percentile, and obesity if their BMI is ≥95th percentile for age and sex [426]. Extreme obesity in this age group is typically defined as a BMI ≥120% of the 95th percentile or ≥35 kg/m^2^ [426]. As a consequence, the World Health Organisation recommends that for children younger than 2 years, a sex-specific weight for recumbent length ≥97.7th percentile be used [426], and clinicians are advised to calculate, plot, and review a child’s or adolescent’s BMI percentile at least annually [426].

Although BMI remains a convenient and widely used screening tool, it has well-recognised limitations. According to the Clinical Practice Guideline published in 2023 by the American Academy of Pediatrics, it does not directly measure body fat or composition, leading to an under- or over-detection of excess adiposity in certain ethnic groups or individuals with higher lean mass [426,428,429]. Moreover, BMI z-scores are less reliable in cases of severe obesity, because the reference population had insufficient data which could result in incorrect extrapolations, or because compression effects observed at extremely high BMI values [429]. To address these limitations, extended BMI reference charts have been developed, incorporating recent population data to improve the accuracy of assessments beyond the 95th percentile [429]. The utility of BMI is further constrained by its variable correlation with comorbidities across different racial and ethnic groups, as well as by increased muscle mass [426]. Consequently, BMI is considered a specific but less sensitive metric for identifying elevated body adiposity in children, often failing to detect excessive adiposity in a considerable proportion of this population [428]. A more comprehensive body composition analysis may be required to address these limitations [430].

From a therapeutic perspective, bariatric surgery is currently recognised as the only proven effective treatment for extreme obesity that provides substantial and sustained weight loss and significant improvement or resolution of associated comorbidities [426,429]. For adults suffering from severe obesity, bariatric surgery is a recognised and established therapeutic approach, often employed in conjunction with behavioural interventions and pharmacotherapy [431]. Standard bariatric procedures, such as laparoscopic Roux-en-Y gastric bypass and vertical sleeve gastrectomy, have demonstrated long-term reductions in BMI and significant improvements in conditions like T2DM, hypertension, dyslipidemia, and various cardiovascular risk factors. For example, bariatric surgery has been shown to reverse T2DM, enhance glucose regulation in non-diabetics, resolve sleep apnea, and improve nonalcoholic steatohepatitis and severe arthropathy [426].

Emerging data also highlight the benefits of bariatric surgery in adolescents with severe obesity, leading to significant and sustained weight loss and amelioration of comorbidities [429,431]. Early surgical intervention in adolescents may appear particularly advantageous, given its association with higher remission rates for certain cardiometabolic risk factors compared to adults [429]. While obesity may persist even after surgery in some extremely obese individuals, the marked improvement in associated health conditions and quality of life underscores the profound impact of these interventions [426,431]. For instance, the Teen-LABS multicentre study reported an average weight loss of 27% three years after surgery, with marked improvements in metabolic outcomes [431,432]. Another study showed a 38% decline in BMI one year after RYGB in extremely obese adolescents [426]. Beyond weight reduction, bariatric surgery in adolescents has been associated with substantial improvement or complete remission of multiple obesity-related comorbidities. Data from the Teen-LABS study demonstrated that, three years post-surgery, remission occurred in 95% of adolescents with T2DM, 76% with prediabetes, 74% with hypertension, and 66% achieved normalisation of lipid profiles. [426]. Furthermore, significant enhancements were observed in glucose homeostasis among non-diabetic individuals, alongside heightened insulin sensitivity and secretion, resolution of sleep apnea, and amelioration of nonalcoholic steatohepatitis and severe arthropathy [426]. Cardiovascular risk factors were also observed to be improved, along with an elevated adiponectin level and a reduction in the levels of inflammatory markers, including IL-1, IL-8, CRP, and TNF-α [426].

Further supporting evidence from Australian studies demonstrates a mean weight reduction of 34.6 kg in the surgical group at two years post-surgery in adolescents aged 14–18, which was greater than that in the lifestyle program [431]. Adolescent patients undergoing gastric bypass also exhibited significantly higher rates of diabetes remission compared to adults [426,429].

Overall, bariatric surgery markedly improves metabolic health, systemic inflammation, and quality of life in individuals with extreme obesity. Although in some extremely obese individuals obesity may persist even after surgery, the marked improvement in associated health conditions and quality of life underscores the profound impact of these interventions [426,431].

### 7.6. Shifting Paradigms

Understanding obesity as a condition of epigenetic dysregulation reinforces the need for early prevention strategies that address not only caloric imbalance but also environmental and psychosocial factors that can influence the epigenome from conception onwards. Tailoring interventions aimed at modifying epigenetic susceptibility factors may offer a powerful tool to reduce the transgenerational perpetuation of obesity and its damaging health consequences. Overall, obesity-induced hormonal dysfunctions reflect a complex network of disrupted feedback loops, neuroendocrine rewiring, and inflammatory interference. Therapeutic strategies targeting weight loss, insulin sensitivity, inflammation control, and even microbiota modulation have shown promising results in restoring endocrine balance and reproductive capacity [115]. Collectively, these findings demonstrate that obese adipose tissue undergoes an immunological transformation, evolving from a passive energy reservoir into an active immune participant in the development of metabolic diseases, that perpetually releases inflammatory mediators driving insulin resistance, cardiovascular disease, and hepatic steatosis [111]. This conceptual shift positions adipose tissue as a central regulator of systemic immunometabolic homeostasis, intricately connected with chronic inflammation and metabolic health. Targeting specific components of this immunometabolic network provides new opportunities for the development of innovative therapeutic interventions. Addressing this inflammatory burden is crucial for achieving real comprehensive and personalised treatment in the management of the global obesity epidemic [111,112,333,433]. Complementary interventions derived from epigenetic research, together with lifestyle modifications, targeted pharmacological approaches, and microbiota-based therapies, are summarised in Table 6. These approaches collectively reflect the paradigm shift from weight-centric management towards restoring systemic metabolic and inflammatory equilibrium, highlighting the multifactorial nature of effective obesity treatment

## 8. Conclusions and Perspectives

Obesity is now recognised as a chronic, systemic disease that transcends the simplistic view of excess fat accumulation. It represents a state of complex metabolic and inflammatory disequilibrium, in which adipose tissue acts as an active endocrine and immune organ driving persistent low-grade inflammation, termed metaflammation. This inflammatory network disrupts insulin signalling, promotes hepatic steatosis and cardiovascular dysfunction, and exerts profound effects on neuroendocrine and reproductive axes.

Importantly, the cross-talk between adipose tissue, immune cells, and the gut microbiota sustains and amplifies this metaflammatory loop. Gut dysbiosis, characterised by altered microbial composition, increased intestinal permeability, and metabolic endotoxemia, contributes to systemic inflammation and energy imbalance through the gut-brain and gut–liver axes. Parallel to these processes, epigenetic modifications, including DNA methylation, histone changes, and ncRNAs, translate environmental and dietary factors into durable molecular signatures, perpetuating susceptibility to metabolic dysfunction across generations.

This integrated understanding of obesity as an immunometabolic disorder underscores the need to move beyond calorie-centric paradigms. Therapeutic strategies should target the restoration of metabolic–immune homeostasis by combining nutritional and behavioural interventions with microbiota modulation, anti-inflammatory therapies, and precision medicine approaches tailored to individual genetic and epigenetic backgrounds.

Future research should aim to disentangle causal relationships within this interconnected network, identify biomarkers of metaflammation that can predict disease trajectory, and develop interventions capable of reprogramming immune and metabolic memory. Addressing obesity, therefore, requires a multidisciplinary, translational approach that integrates metabolic, microbial, and immunological perspectives to achieve durable and systemic health restoration.

## Figures and Tables

**Table 1:** Obesity as a systemic dysfunction.

Affected System	Main Mechanism	Associated Effects	Experimental Model	Principal Findings
Neuroendocrine	Leptin resistance (impaired transport across blood–brain barrier (BBB), defective receptor signalling, suppressor of cytokine signalling 3 (SOCS3) activation, hypothalamic inflammation (microglial activation, astrogliosis, cytokine secretion), ghrelin dysregulation (decreased active acyl-ghrelin), hormonal imbalances (e.g., cortisol elevation, altered adipokines), impaired glucose-sensing in hypothalamus, altered neuroendocrine axes [17,19]	Appetite dysregulation (hyperphagia), impaired satiety signalling, reduced fertility (anovulation, impaired spermatogenesis) [20,21,22], hypogonadism [23], disrupted pubertal timing, increased central fat accumulation, PCOS, impaired glucose homeostasis [24,25], altered energy expenditure [26,27], cognitive and mood disorders [28]	Cohort study of 99 obese subjects undergoing Roux-en-Y gastric bypass surgery [29].	The study by Ekberg et al. [29] aimed to identify and compare clinical biomarkers for insulin sensitivity and other parameters in predicting the normalisation of HbA1c after RYGB surgery in subjects with abnormal glucose levels. While specific predictors were not detailed in the available excerpts, the research focused on these factors post-surgery [29].
Immune	Chronic low-grade inflammation (metaflammation) due to nutritionally overloaded metabolic cells, M1 macrophage infiltration and polarization (driven by altered lipid metabolism, increased glycolysis, hypoxia-inducible factor-1α (HIF-1α) activation), Nucleotide-binding oligomerization domain, Leucine-rich repeat and Pyrin domain containing pyrin domain-containing protein—3 (NLRP)-3) inflammasome activation (by oxidized LDL, cholesterol crystals, hyperglycaemia, free fatty acids, excess ATP, reactive oxygen species), dysfunctional adipocyte signalling, immune cell infiltration into adipose tissue (T lymphocytes, NK cells, mast cells, dendritic cells), persistent epigenetic reprogramming (trained immunity) [30,31,32,33,34,35,36,37,38,39,40]	Insulin resistance (impaired insulin signalling via serine phosphorylation of IRS-1, activation of JNK, IKK, PKR) [41,42,43,44,45,46], increased pro-inflammatory cytokine secretion (TNF-α, IL-1β, IL-6), enhanced inflammatory gene expression, exacerbation of T2DM, endothelial dysfunction, systemic chronic low-grade inflammation [47,48,49,50], impaired adipose tissue function.	Gnotobiotic mice stimulated by dysbiotic gut microbiota transplant from a genetically obese child [51]. Obese mice and humans (mechanism studied by Fang et al. [52]).	Deng et al. [51] observed upregulated pro-inflammatory genes in the colon and liver, and altered glucagon-like peptide 1/insulin receptor signalling. Fang et al. [52] identified that obesity promotes a leaky gut, inflammation, and pre-diabetes by lowering gut microbiota that metabolise ethanolamine.
Metabolic	Insulin resistance, lipotoxicity (excess free fatty acid release, accumulation of diacylglycerol and ceramides), reduced short-chain fatty acids from dysbiosis, mitochondrial dysfunction, endoplasmic reticulum stress, glucose-fatty acid cycle imbalance [18,19,53,54,55,56,57,58,59,60,61,62,63,64,65]	T2DM, hepatic steatosis [66,67,68,69], dyslipidemia, metabolic syndrome, impaired glucose uptake in muscle [18], altered energy metabolism, increased oxidative stress, impaired β-cell function, cardiovascular disease [70]	Gnotobiotic mice stimulated by dysbiotic gut microbiota transplant from a genetically obese child [51]. Obese mice (diet-induced obesity) treated with *Akkermansia muciniphila* [71]. Cohort study of 99 obese subjects undergoing Roux-en-Y gastric bypass surgery [29]. Obese mice and humans (mechanism studied by Fang et al. [52]).	Deng et al. [51] found lipid and cholesterol accumulation in the liver and decreased insulin receptor signalling. Depommier et al. [71] demonstrate that *Akkermansia muciniphila* improves metabolic parameters, enhances fatty acid oxidation, and improves glucose homeostasis by increasing mono-palmitoyl-glycerol (1-PG), a PPARα agonist. Ekberg et al. [29] aimed to predict HbA1c normalisation post-surgery. Fang et al. [52] proposed that reduced ethanolamine-metabolising microbiota contribute to leaky gut and metabolic dysfunction.
Epigenetic	DNA methylation (e.g., at the level of LEP, adiponectin (ADIPOQ), Peroxisome Proliferator-Activated Receptor γ(PPAR-γ), PGC-1a genes), microRNA (miRNA) deregulation (e.g., miR-27a, miR-143, miR-146a), histone modifications, early metabolic programming due to maternal hypernutrition/gestational inflammation, environmental chemical exposure [4,9,10,72,73,74,75,76,77,78,79,80,81,82,83,84,85,86,87,88]	Transgenerational risk transmission of obesity and metabolic disorders, altered gene activity and expression patterns in adipogenesis, lipid metabolism, insulin signalling, and inflammatory responses [9,10,85,86], potential for biomarkers and therapeutic targets [75,80,81], influence on susceptibility to diseases [10], reversible with lifestyle changes [10,82]	Gnotobiotic mice stimulated by dysbiotic gut microbiota transplant from a genetically obese child [51].	Deng et al. [51] investigated miRNA-gene regulatory networks in response to dysbiotic gut microbiota, revealing altered gene expression patterns relevant to host phenotype changes and demonstrating molecular changes before body fat changes.
Microbiota	Gut microbiota dysbiosis (reduced diversity, altered *Firmicutes*/*Bacteroidetes* ratios), increased intestinal permeability (“leaky gut”) allowing bacterial lipopolysaccharides translocation, metabolic endotoxemia, altered bile acid metabolism, modulation of gut hormones (ghrelin, glucagon-like peptide-1 (GLP-1), Peptide tyrosine (PYY) [11,52,71,89,90,91,92,93,94,95,96,97,98,99,100,101,102,103,104,105,106]	Systemic inflammation, persistent obesity, reduced short-chain fatty acid production, impaired glucose homeostasis, insulin resistance, increased energy harvest from diet, activation of pro-inflammatory pathways, hepatic steatosis [107], altered central control of appetite and metabolic processes, influence on neuroinflammation via gut–brain axis, and increased oxidative stress.	Metagenomic analysis of human gut microbiome [108]. Gnotobiotic mice stimulated by dysbiotic gut microbiota transplant from a genetically obese child [51]. Obese mice (diet-induced obesity) treated with *Akkermansia muciniphila* [71]. Obese mice and humans (mechanism studied by Fang et al. [52]). Microbial manipulation in mouse CRC models (mechanisms studied by Zheng et al. [109])	Das et al. [108] identified specific bacterial enzymes and pathways involved in bile salt biotransformation, which in turn influence host lipid and cholesterol metabolism. Deng et al. [51] found that dysbiotic gut microbiota transplantation led to molecular changes, which were observed as early as the second week, two weeks before changes in body fat occurred. Depommier et al. [71] showed that *A. muciniphila* influences lipid mediators (like 1-PG) to improve metabolic health. Fang et al. [52] suggest that obesity leads to leaky gut and metabolic dysfunction by reducing ethanolamine-metabolising gut microbiota.

**Table 2:** NLRP3 inflammasome and metabolic implications.

Trigger Factor	Target Tissue	Metabolic Effect	Experimental Model	Principal Findings	References
LPS	Adipocytes, Macrophages	IL-1β, IL-18 activation; systemic inflammation	Murine models (C57BL/6J mice) [167], rat models [168], THP-1 macrophages [169]. In vitro studies on bone marrow derived macrophages and 3T3-L1 adipocytes [170,171].	LPS induces an inflammatory cascade in macrophages and adipocytes via TLR4, leading to the production of inflammatory cytokines like TNF-α, IL-1β, and iNOS [169]. In mice, LPS causes a marked decrease in lipin-1 mRNA in adipose tissue [170]. LPS stimulation of peritoneal and bone marrow-derived macrophages decreases ApoE mRNA levels [172]. Intravascular infusion of free fatty acids activates NF-kB in adipose tissue, involving TLR4 in macrophages and adipocytes [167].	[13,34,111,167,169,170,171,172,173]
Adipocyte hypoxia	Adipose tissue	HIF-1α and inflammasome activation	Mouse models of obesity (ob/ob and dietary obese mice) [174], adipocyte-specific Hif-1α KO mice [175].	Hypoxia in expanding white adipose tissue leads to increased HIF-1α expression [174]. HIF-1α in adipocytes regulates lysophosphatidylcholine metabolism and is necessary for homocysteine-induced insulin resistance [176]. Adipocyte-specific HIF-1α deletion prevents adipose tissue inflammation and insulin resistance induced by a high-fat diet in mice [177]. Hypoxia triggers ROS production, ER stress, and inflammatory responses [174].	[33,151,174,175,176,177,178,179,180,181]
Reduced SCFA	Intestine, Adipose tissue	Leaky gut, dysbiosis; impaired glucose metabolism	Fructose-fed C57BL/6N mice [182]. Studies linking gut microbiota metabolites to intestinal and systemic health [183].	Reduced SCFAs lead to intestinal epithelial barrier impairment and gut dysbiosis in fructose-fed mice [182]. SCFA butyrate is effective in alleviating diet-induced obesity [11]. Microbiota-derived SCFAs, such as propionate, activate ileal free fatty acid receptor 2 to lower hepatic glucose production [184].	[11,182,183,184,185,186,187,188,189]
Molecular byproducts of ageing, physical inactivity, Western diet	Systemic	Systemic chronic low-grade inflammation	Human cohorts and animal models studying inflammaging and metabolic stress [190,191].	Western societies’ lifestyle and excess calorie consumption can aggravate age-related inflammatory responses, known as metaflammation [191]. The NLRP3 inflammasome is centrally involved in recognizing triggers during physiological aging and metabolic stress [191]. A sedentary lifestyle and high-fat diet can lead to obesity and chronic low-grade inflammation [192].	[35,39,190,191,192,193,194,195,196]
Oxidised low-density lipoprotein	Macrophages	Increased inflammation	Animal models of atherosclerosis (e.g., cholesterol-fed rabbits, LDL-receptor-deficient mice) [197,198], human THP-1 macrophages [199].	OxLDL is taken up by macrophages, leading to cholesterol accumulation and the formation of foam cells, which contributes to atherogenesis [198,200]. OxLDL can induce alternative macrophage phenotypes, affecting cytokine production [199]. OxLDL enhances pro-inflammatory responses of M2 macrophages, shifting them towards a pro-inflammatory profile [201].	[35,197,198,199,200,201,202,203,204,205,206,207,208]
Cholesterol crystals	Macrophages	Increased inflammation	ApoE-knockout mice fed a high-cholesterol diet [209], human macrophages [210].	Cholesterol crystals in atherosclerotic lesions drive IL-1β production in macrophages, and this effect is lost in NLRP3- and ASC-deficient macrophages [209]. Cholesterol crystals activate the NLRP3 inflammasome in human macrophages, linking cholesterol metabolism and inflammation [210]. NLRP3 inflammasomes are required for atherogenesis and activated by cholesterol crystals [211].	[36,192,209,210,211,212,213]
Hyperglycaemia	THP-1-derived macrophages, 3T3-L1 mature adipocytes, human adipose tissue, pancreatic islets	Impaired glucose metabolism; insulin resistance; increased IL-1β and IL-18 production	THP-1-derived macrophages, 3T3-L1 adipocytes, human adipose tissue [34]. Bone marrow-derived macrophages from diabetic mice [214].	Hyperglycaemia stimulates NLRP3 inflammasome activation in various cell types [34]. High glucose treatment increases IL-1β expression in macrophages [215]. Hyperglycaemia itself can induce a mixed M1/M2 cytokine profile in primary human monocyte-derived macrophages, supporting diabetes-associated inflammation [216].	[34,35,159,169,171,214,215,216,217,218,219]
Free fatty acids, Ceramides, Excess ATP	Adipose tissue, Systemic	Insulin resistance; pro-inflammatory response; contributes to metabolic syndrome	Mouse liver cell lines [220], various cell types (macrophages, adipocytes) [221], rodent models [222].	Palmitate activates NLRP3, enhances ROS generation, and can suppress insulin-induced Akt phosphorylation, suggesting FFA-mediated insulin resistance [220]. Saturated fatty acids induce ceramide formation, which are potent antagonists of insulin action [222]. In both macrophages and adipocytes, ceramides activate the NLRP3 inflammasome [221]. Fatty acid-induced NLRP3-ASC inflammasome activation interferes with insulin signalling [166].	[33,34,35,161,166,218,220,221,222,223,224,225,226,227]
Reactive oxygen species	Systemic	Activates NLRP3 inflammasome; leads to insulin resistance and metabolic syndrome	Rat hepatocytes [228], human studies on diabetic complications [229].	ROS drives fructose-mediated hepatic inflammation and lipid accumulation through NLRP3 inflammasome activation [228]. Elevated mitochondrial ROS in myeloid cells of T2DM patients are associated with increased production of inflammasome-dependent cytokines IL-1β and IL-18 [159]. NOD2 activation enhances ROS production in skeletal muscle cells, contributing to mitochondrial dysfunction and insulin resistance [230].	[33,39,159,192,228,229,230,231,232]
Metabolic insults	Adipose tissue	Exacerbates insulin resistance; promotes adipose tissue dysfunction	Human and animal studies [34,232].	Metabolic insults, including SFAs, pro-inflammatory adipokines, hyperglycaemia, and endotoxemia, are major stimuli for NLRP3 inflammasome activation in adipose tissue [34]. NLRP3 inflammasome activity in adipose tissues positively correlates with obesity and its metabolic complications [34].	[34,38,39,63,206,211,232,233]
Danger signals from stressed/dying adipocytes	Adipocytes	Activation of NLRP3-ASC inflammasome; interferes with insulin signalling	In vitro studies on adipocytes [234].	Endogenous danger signals activate NLRP3 inflammasome, leading to processing and secretion of IL-1β and IL-18 [235]. Released fatty acids induce NLRP3-ASC inflammasome activation, interfering with insulin signalling and reducing insulin sensitivity [166]. Adipocyte death triggers a pro-inflammatory response and metabolic activation of resident macrophages [234].	[31,33,63,137,166,234,235,236,237]

**Table 3:** Microbiota influence on bile acid metabolism in obesity.

Microorganism	Bile Acid Metabolism	Metabolic Effect	Experimental Model	Principal Findings	References
*Bacteroidetes* (phylum)	Possesses bile salt hydrolase activity, deconjugating primary bile acids	Affects host lipid and cholesterol metabolism, influences weight gain	Gnotobiotic mice monocolonized with wild-type or BSH-deleted *Bacteroides thetaiotaomicron* strains [254]. Germ-free and conventionally raised murine models with bacterial BSH expression [265]. High-fat diet-induced hyperlipidemic rats treated with *Bacteroides vulgatus* [266].	*Bacteroides thetaiotaomicron* with BSH activity selectively modulated levels of tauro-β-muricholic acid [254]. High-level BSH expression reduced host weight gain, plasma cholesterol, and liver triglycerides in mice [265]. *Bacteroides vulgatus* ameliorated serum lipid profiles and systemic inflammation in hyperlipidemic rats by changing bile acid metabolism [266]. BSH mediates a microbe-host dialogue regulating host lipid and cholesterol metabolism and weight gain [255].	[250,254,255,265,266,267,268,269,270]
*Firmicutes* (phylum)	Some species involved in the formation of secondary allo-bile acids can be associated with increased energy harvesting	Altered bile acid profile; influences energy balance and contributes to obesity	Metagenomic and phylogenetic analyses of human stool samples [271]. Studies on primary bile acid (cholic acid) influence on gut microbiota composition [272].	Firmicutes generate secondary allo-bile acids (allo-DCA and allo-LCA) through novel enzymes (BaiA1, BaiP, BaiJ) [271]. Increased primary bile acid levels can shift the gut microbiome towards Firmicutes, particularly *Clostridium* cluster XIVa, leading to increased deoxycholic acid production [272].	[89,90,108,265,271,272,273,274,275,276,277,278]
*Clostridium, Enterococcus, Lactobacillus, Bifidobacterium, Ruminococcus, Xanthomonas, Eubacterium*	Contribute to the transformation of primary into secondary bile acids	Altered secondary bile acid profile, influencing host metabolic pathways	Rodent models fed high-fat diets [279]. Human metagenome analysis [280]. Studies identifying bile acid-converting bacteria [281].	*Clostridium scindens* and *Clostridium hylemonae* were abundant in obesity-prone rodents, modifying BA metabolism and promoting obesity [279]. Species within *Clostridium* and *Eubacterium* generate secondary bile acids that can modulate adiposity via FXR or TGR5 signalling [280]. Gut bacteria that convert primary BAs to secondary BAs belong to a limited number of species, mainly Clostridiales [281].	[270,273,279,280,281,282,283,284]
*Akkermansia muciniphila*	Indirectly influences bile acid-related metabolic health through modulation of the endocannabinoid system, body weight, and food intake regulation	Associated with improved metabolic parameters; indirectly linked to bile acid-influenced metabolic health	High-fat diet-fed mice administered *A. muciniphila* [285,286,287]. Obese and type 2 diabetic mice [286].	*A. muciniphila* administration reversed high-fat diet-induced metabolic disorders, including fat-mass gain, metabolic endotoxemia, adipose tissue inflammation, and insulin resistance, by increasing intestinal endocannabinoids [286]. Oral transfer of live *A. muciniphila* ameliorated obese and diabetic phenotypes, reduced metabolic endotoxemia, host adiposity, and improved glucose metabolism [287]. It influences endocannabinoid concentrations in the ileum, such as 2-AG, associated with improved metabolic dysfunctions [285].	[90,285,286,287,288,289,290,291,292,293,294]
General dysbiotic state	Decreased beneficial microbiota and increased opportunistic pathogens (e.g., Bacilli, *Enterobacteriales*, *Streptococcaceae*, *Veillonella*, *Lactobacillales* increase; *Clostridia*, *Clostridiales* decrease)	Correlates with higher bile acid levels; reflects or contributes to altered bile acid metabolism in disease states, including those related to obesity	Human cohort studies of obese and metabolically unhealthy individuals [279]. High-fat diet-fed mice [102,264].	Unhealthy obese subjects had a significantly lower proportion of secondary bile acids compared to primary ones, suggesting altered BA composition is involved in different metabolic states of obesity [279]. A high-fat diet caused rapid increases in the intestinal BA pool and altered microbial composition, driving modifications of BA and microbiota compositions that trigger metabolic disorders [102]. Long-term HFD feeding decreased hepatic and serum BA levels and impaired gut microbiota, with higher bile salt hydrolase activity in ileal microbes [264].	[101,102,103,264,272,279,283]

**Table 4:** Epigenetic modifications related to obesity.

Type of Modification	Trigger Factor	Consequences	Experimental Model	Principal Findings	References
DNA methylation	High-fat maternal diet	Increased T2DM risk in offspring; altered gene activity and changes in DNA methylation patterns predisposing to obesity and related comorbidities; may be transgenerationally inherited	Pregnant mice or rats fed a high-fat diet [173,326,327,328]. Studies on intrauterine exposure to obesogenic environments.	Maternal high-fat diet induces DNA methylation changes in offspring’s brown adipose tissue, impacting obesity progression [329] and contributes to glucose intolerance [327]. Leads to lasting epigenetic alterations, programming metabolic pathways and adipogenesis. Gestational high-fat diet impaired demethylation of PPARα in offspring’s liver, inducing obesity [173]. Maternal overnutrition alters DNA methylation in early life, predisposing offspring to metabolic diseases [326].	[173,326,327,328,329,330,331,332,333,334,335]
DNA methylation	Environmental factors	Altered gene activity, changes in DNA methylation patterns predisposing to obesity and related comorbidities; risk of dyslipidemia; potential for biomarkers and therapeutic targets	Human cohorts (e.g., obese vs. lean individuals) [336,337,338], animal studies on obesogenic environments [84,339].	Obese individuals exhibit distinct epigenetic signatures. Environmental factors link to altered gene activity and obesity phenotypes [337]. Differential methylation of genes like PPARγ, LEP, and FTO correlates with adipocyte differentiation and insulin resistance. Global hypomethylation in subcutaneous adipose tissue and leukocytes of obese individuals has been observed [336].	[10,84,336,337,338,339,340,341,342]
miRNA deregulation	Chronic inflammation	Pro-inflammatory gene expression, dysregulation of central lipid and glucose metabolism, impaired target gene expression in obese adipose tissue, insulin resistance, altered lipid metabolism; potential for biomarkers and therapeutic targets	Human primary mature adipocytes and macrophages [343], pediatric cohorts with severe obesity [344], 3T3-L1 adipocytes [345].	Altered expression of miRNAs (e.g., miR-27a, miR-143, miR-146a) contributes to insulin resistance, adipose tissue inflammation, and impaired lipid metabolism. Circulating miRNAs (e.g., miR-34a, miR-122, miR-192) are associated with obesity-related inflammation and metabolic disease in pediatric patients [344]. Downregulation of miR-320 alleviates endoplasmic reticulum stress and inflammatory response in 3T3-L1 adipocytes [345].	[74,80,343,344,345,346,347,348,349,350,351,352,353,354]
miRNA deregulation	Gut microbiota dysbiosis	Regulation of white adipose tissue inflammation and obesity	Human cohorts comparing obese and eutrophic individuals [355], mouse models of high-fat diet-induced obesity [356], gnotobiotic mice stimulated by dysbiotic gut microbiota transplant [51].	Host-secreted miRNAs regulate the gut microbiota, and the gut microbiota affects the host via inducing special miRNA expression [357]. Circulating miRNAs and gut microbiota composition show novel interactions in human obesity [355]. Dysbiotic gut microbiota transplantation in mice led to miRNA deregulation and altered gene expression impacting host phenotype [51].	[51,292,352,355,356,357,358,359,360,361,362]
Histone modifications	Dysbiosis and oxidative stress	Altered transcription of metabolic genes, impaired energy metabolism, changes in adipogenesis	Mouse models of high-fat diet [363], studies on gut dysbiosis [364], intestinal epithelial cells [365].	Gut dysbiosis can induce excessive production of reactive oxygen species, leading to inflammation and epigenetic alterations [364]. Microbiota-derived short-chain fatty acids can cause epigenetic imprinting in utero [366]. Diet-microbiota interactions mediate global histone acetylation and methylation in multiple host tissues [367]. HFD induces histone modification in adipose tissues, activating genes related to lipogenesis, energy metabolism, and inflammation [363].	[206,342,363,364,366,367,368,369,370,371]
Histone modifications	Environmental factors	Influence on gene expression without altering the DNA sequence	Human and animal studies on environmental exposures [84,339].	Histone modifications mediate the interaction between genetic predisposition and environmental exposures. Environmental factors, including nutrition, inflammation, hypoxia, and physical activity, can induce epigenetic changes [372]. Chromatin modifications are associated with the progression of diabetes and obesity [373].	[10,82,84,206,339,372,373,374]
General epigenetic changes	Multifactorial	Influence susceptibility to diseases like obesity, contribute to disordered energy metabolism; can be reversed with lifestyle changes; provide insights into pathophysiological processes for prevention and treatment; potential for new therapeutic approaches	Weight loss interventions (lifestyle, pharmacotherapy, bariatric surgery) [88,375,376,377,378,379,380].	Epigenetic phenomena are dynamic and reversible with intensive lifestyle changes [82]. Weight loss interventions (dietary changes, exercise, surgical interventions) can reverse epigenetic modifications, including methylation patterns of leptin and adiponectin genes [88]. Bariatric surgery can lead to significant improvements in methylation of genes regulating insulin sensitivity and inflammatory responses [379,381].	[10,82,88,206,372,375,376,377,378,379,380,381,382,383,384,385,386,387]

**Table 5:** Overview of major obesity precision medicine initiatives.

Intervention	Main Target	Systemic Effects	Examples/References
targeting metaflammation	Chronic low-grade inflammation, oxidative stress, NLRP3 inflammasome	Ameliorates metaflammation and its metabolic consequences, restores adipose homeostasis, mitigates obesity-associated cardiometabolic complications	Novel anti-inflammatory compounds, gut microbiota modulators, NLRP3 inhibitors, inhibiting inflammatory responses by neutralising cytokines/chemokines, deleting Toll-like receptors, blocking neutrophil recruitment [5]
microbiota modulation	Gut microbiota composition and function, intestinal barrier integrity, microbial metabolites	Mitigates metaflammation, improves metabolic health, reduces intestinal low-grade inflammation, improves gut barrier integrity, ameliorates metabolic balance, promotes weight loss, improved glucose homeostasis and satiety	Dietary interventions (fermentable fibres, prebiotics) [415], Probiotics (e.g., *Lactobacillus gasseri*) [93,416], Faecal Microbiota Transplantation [93,417,418], manipulating microbial metabolites [73], *Akkermansia muciniphila* [93,292], *Blautia wexlerae* [419]
precision nutrition and multi-omics	Individual metabolic and microbial profiles, specific biomarkers and pathways implicated in metaflammation, individual’s genetic, environmental, lifestyle characteristics, obesity subtypes/phenotypes	Optimises anti-inflammatory responses, fosters sustained metabolic health, enables individualised prevention and treatment strategies, enhances treatment effectiveness and tolerability	Integration of omics data (genomic, epigenomic, proteomic, metabolomic, microbiomic profiling) [90,119,390,403,404,405,420], DNA methylation, metabolomics, gut microbiome data to predict weight loss [421]
targeting macrophage dynamics	Macrophage function, adipocytes, inflammation within adipose tissue, ceramide accumulation, inflammatory kinases	Mitigates obesity-associated metaflammation, improves metabolic outcomes, reduces inflammation, improves adipose tissue health, restores catecholamine sensitivity, reverses metabolic dysfunctions associated with obesity	Targeting M1 to M2 macrophage polarisation [32,147,246,412,422,423], ceramide synthesis inhibitors, Amlexanox, Metformin [31], Thiazolidinediones [31], adipose tissue-targeting ultra-small hybrid nanoparticles [424], Interferon Tau [422]

**Table 6:** Systemic effects of therapeutic interventions.

Intervention	Main target	Systemic Effects	References
High-fibre, polyphenol-rich diet	gut microbiota	↑ SCFA, ↓ Endotoxemia, ↓ Inflammation	[415,434]
Regular physical activity	adipose tissue, HPG/HPA axis	↑ LEP sensitivity, ↓ Inflammation	[435,436,437,438]
IL-1β inhibitors	systemic inflammation	↓ Inflammatory markers, better glycaemic control	[439,440,441,442,443,444,445,446]
weight loss surgery	adipose tissue, metabolic organs	↓ Adipokines, improved insulin sensitivity	[29,447,448,449,450,451,452,453,454,455,456,457,458,459,460]
GLP-1 Receptor Agonists	appetite regulation, satiety, gastric emptying, glucose-dependent insulin secretion, glucagon release	Significant weight loss, improved glucose metabolism, enhanced insulin sensitivity, cardiovascular protection, anti-inflammatory and anti-atherogenic actions, improved blood pressure and lipid profile	[461,462,463,464,465,466,467,468,469,470,471,472,473,474,475]
microbiota modulation	gut microbiota composition and function, intestinal barrier integrity	↓ Systemic inflammation, improved metabolic signalling, improved metabolic balance, weight loss, increased SCFA production, reduced metabolic endotoxemia	[415,434]
targeting macrophage dynamics	macrophage function, adipocyte clearance, inflammatory pathways	↓ Adipose tissue inflammation, improved metabolic outcomes, restored M2 macrophage phenotype, improved insulin sensitivity, inhibition of ceramide synthesis	[246,348,422,476]
leptin sensitising/adiponectin-based therapies	leptin resistance, adiponectin signalling	Overcome LEP resistance, improved insulin resistance, positive effects on fatty liver and other organ disturbances, enhanced energy expenditure, reduced weight gain	[447] (for LEP to adiponectin ratio), [437] (for adipokine regulation by exercise)
epigenetic modulation	epigenetic modifications	Reversal of unfavourable gene expression patterns, improved metabolic profiles, restored energy balance, reduced inflammation, potential for transgenerational impact reversal	[447,448,449,450,451,452,453,454,455,456,457,458,459,460,461,462,463,464,465,466,467,468,469,470,471,472,473,474,475,476,477,478,479,480]

↑—increased level ↓—reduced level.

## Data Availability

No new data were created or analyzed in this study. Data sharing is not applicable to this article.
